# Recovery of the Peptidoglycan Turnover Product Released by the Autolysin Atl in *Staphylococcus aureus* Involves the Phosphotransferase System Transporter MurP and the Novel 6-phospho-*N*-acetylmuramidase MupG

**DOI:** 10.3389/fmicb.2018.02725

**Published:** 2018-11-16

**Authors:** Robert Maria Kluj, Patrick Ebner, Martina Adamek, Nadine Ziemert, Christoph Mayer, Marina Borisova

**Affiliations:** ^1^Microbiology/Biotechnology, Department of Biology, Interfaculty Institute of Microbiology and Infection Medicine, University of Tübingen, Tübingen, Germany; ^2^Microbial Genetics, Department of Biology, Interfaculty Institute of Microbiology and Infection Medicine, University of Tübingen, Tübingen, Germany

**Keywords:** peptidoglycan recycling, cell wall turnover, *Staphylococcus aureus*, Atl autolysin, peptidoglycan hydrolases, 6-phosphomuramidase, exo-*N*-acetylmuramidase, MurNAc-GlcNAc

## Abstract

The peptidoglycan of the bacterial cell wall undergoes a permanent turnover during cell growth and differentiation. In the Gram-positive pathogen *Staphylococcus aureus*, the major peptidoglycan hydrolase Atl is required for accurate cell division, daughter cell separation and autolysis. Atl is a bifunctional *N*-acetylmuramoyl-L-alanine amidase/endo-β-*N*-acetylglucosaminidase that releases peptides and the disaccharide *N*-acetylmuramic acid-β-1,4-*N*-acetylglucosamine (MurNAc-GlcNAc) from the peptido-glycan. Here we revealed the recycling pathway of the cell wall turnover product MurNAc-GlcNAc in *S. aureus*. The latter disaccharide is internalized and concomitantly phosphorylated by the phosphotransferase system (PTS) transporter MurP, which had been implicated previously in the uptake and phosphorylation of MurNAc. Since MurP mutant cells accumulate MurNAc-GlcNAc and not MurNAc in the culture medium during growth, the disaccharide represents the physiological substrate of the PTS transporter. We further identified and characterized a novel 6-phospho-*N*-acetylmuramidase, named MupG, which intracellularly hydrolyses MurNAc 6-phosphate-GlcNAc, the product of MurP-uptake and phosphorylation, yielding MurNAc 6-phosphate and GlcNAc. MupG is the first characterized representative of a novel family of glycosidases containing domain of unknown function 871 (DUF871). The corresponding gene *mupG* (*SAUSA300_0192*) of *S. aureus* strain USA300 is the first gene within a putative operon that also includes genes encoding the MurNAc 6-phosphate etherase MurQ, MurP, and the putative transcriptional regulator MurR. Using mass spectrometry, we observed cytoplasmic accumulation of MurNAc 6-phosphate-GlcNAc in Δ*mupG* and Δ*mupGmurQ* markerless non-polar deletion mutants, but not in the wild type or in the complemented Δ*mupG* strain. MurNAc 6-phosphate-GlcNAc levels in the mutants increased during stationary phase, in accordance with previous observations regarding peptidoglycan recycling in *S. aureus*.

## Introduction

*Staphylococcus aureus* is a small, spherical bacterium (∼1 μm diameter) belonging to the phylum firmicutes that can cause life-threatening infections due to the emergence of multi-drug resistance (Hiramatsu et al., 2014). As a Gram-positive bacterium, *S. aureus* is encased in a thick layer of peptidoglycan (PGN), which maintains cell shape and protects the cells from rupture due to high turgor pressure (Cabeen and Jacobs-Wagner, 2005). The general structure of the PGN is conserved in all eubacteria, consisting of a glycan backbone of two alternating β-1,4-glycosidically linked sugars *N*-acetylmuramic acid (MurNAc) and *N*-acetylglucosamine (GlcNAc) and polypeptides, connected to the D-lactyl moiety of MurNAc, that are partially crosslinked (Vollmer et al., 2008). The PGN polymer is synthesized by glycosyltransferases and transpeptidases [involving shape, elongation, division and sporulation (SEDS) family peptidoglycan synthases and penicillin binding proteins (PBPs)] through polymerization of GlcNAc-β-1,4-MurNAc-peptide building blocks, provided by lipid II precursors (Cho et al., 2016; Meeske et al., 2016). The overall peptidoglycan structure of *S. aureus* is different in some aspects compared to most other bacterial species. Firstly, pentaglycine (Gly_5_) bridges are attached to the ε-NH_2_ group of L-lysine (L-Lys) of the stem tetrapeptide L-alanine-D-isoglutamine-L-Lys-D-alanine (L-ala-D-isoGln-L-Lys-D-Ala) that are extensively cross-linked (degree of crosslinking of about 80%, dependent on the growth phase and growth conditions) with neighboring peptide stems via their carboxyl group of D-Ala (Gally and Archibald, 1993; Litzinger and Mayer, 2010). Secondly, the glycan chains are comparatively short. About 80% of the glycan chains of the mature cell wall have a predominant length of 3–10 disaccharides, the average degree of polymerization is 6 disaccharides, and only about 15% of the glycan chains have a degree of polymerization more than 25 (Boneca et al., 2000).

The coccus-shaped *S. aureus* divides sequentially in three orthogonal planes over three consecutive division cycles (Monteiro et al., 2015). Although elongation-specific cell wall synthesis machinery is absent, the cells elongate before initiation and after completion of the division septum (Monteiro et al., 2015). The final splitting of daughter cells is accompanied by fast reshaping of the flat septum to finally yield two coccoid offspring cells. Thus, the peptidoglycan is remarkably dynamic and it is constantly degraded during the cell cycle of *S. aureus* by peptidoglycan hydrolases (potential autolysins) (Foster, 1995; Ramadurai et al., 1999; Takahashi et al., 2002; Kajimura et al., 2005; Biswas et al., 2006; Frankel et al., 2011; Wheeler et al., 2015; Chan et al., 2016). The major autolysin of *S. aureus*, called Atl, is a multi-domain enzyme, composed of a secretion signal peptide, a propeptide of still unclear function, and two catalytic domains, an N-terminal *N*-acetylmuramoyl-L-alanine amidase as well as a C-terminal endo-β-*N*-acetylglucosaminidase domain, which are interrupted by cell wall binding repeats (Sugai et al., 1995). The enzyme undergoes proteolytic processing to generate two extracellular peptidoglycan hydrolases, a 62 kDa *N*-acetylmuramoyl-L-alanine amidase (Atl_AM_) and a 51 kDa endo-β-*N*-acetylglucosaminidase (Atl_GL_), which localize in a ring-like structure on the cell surface at the septal region, most likely binding to lipoteichoic acids, extending from the cell membrane (Yamada et al., 1996; Götz et al., 2014). Both Atl hydrolase entities can also be secreted and are found in the culture supernatants of some strains (Sugai et al., 1995). Atl functions during cell expansion and division, and it is required for proper daughter cell separation (Sugai et al., 1995; Biswas et al., 2006). Furthermore, Atl is associated with autolysis processes, e.g., during biofilm formation (Biswas et al., 2006; Bose et al., 2012). Besides Atl_GL_, *S. aureus* also possesses three other *N*-acetylglucosaminidases, SagA, SagB, and ScaH, which are important for proper septum formation at the final stage of cell division. SagB was also found to be responsible for shortening of newly synthesized glycan strands to their physiological length, thus ensuring flexibility during the cell elongation process (Wheeler et al., 2015; Chan et al., 2016). Interestingly, the *S. aureus* genome apparently does not encode any *N*-acetylmuramidases, with the exception of two putative lytic transglycosylases, IsaA and SceD (Stapleton et al., 2007), indicating that the processing of glycan strands in this organism involves endo-acting *N*-acetylglucosaminidases, besides peptidoglycan amidases and endopeptidases (Ramadurai et al., 1999; Kajimura et al., 2005; Frankel et al., 2011). The combined activities of these PGN hydrolases generate MurNAc-GlcNAc and peptides as final peptidoglycan turnover products.

It was calculated that during the process of PGN turnover in *S. aureus*, 15% (Wong et al., 1974) or up to 25% (Blümel et al., 1979) of cell wall material fragments are released per generation in the culture medium. However, the ability of Gram-positive bacteria in general to recycle these fragments was questioned for a long time. We recently elucidated that recycling occurs in different Gram-positive bacteria such as *S. aureus*, *Bacillus subtilis*, and *Steptomyces coelicolor* and revealed that the MurNAc 6-phosphate (MurNAc 6P) etherase MurQ (Borisova et al., 2016), responsible for the intracellular conversion of MurNAc 6P to GlcNAc 6-phosphate and D-lactate, is required for this process. In addition, we quantified the intracellular accumulation of MurNAc 6P in the markerless Δ*murQ* mutant, showed that recycling of the MurNAc portion of peptidoglycan occurs predominantly during nutrient limitation within transition and stationary phase (Borisova et al., 2016). Furthermore we discovered that peptidoglycan recycling is essential for bacterial survival in the late stationary phase (Borisova et al., 2016). In *S. aureus* strain USA300, the *murQ* etherase gene (*SAUSA300_0193*) is encoded in an operon together with *SAUSA300_0194*, encoding the enzyme IIB and IIC domains of the MurNAc PTS-transporter MurP, *SAUSA300_0195*, encoding a MurR-like regulator, and *SAUSA300_0192*, encoding a protein with unknown function (Borisova et al., 2016).

We hypothesized that MurNAc-GlcNAc, the product of cell wall cleavage by Atl, rather than MurNAc, might be taken up and recycled in *S. aureus* and proposed a role of the gene *SAUSA300_0192* of unknown function in this process. To prove this hypothesis, we generated markerless gene deletion mutants and investigated the intracellular and extracellular accumulation of specific recycling products in different growth phases by mass spectrometry. Our study showed that MurNAc 6P-GlcNAc accumulates in *SAUSA300_0192* mutant, predominantly during stationary phase, and that this compound is generated by the uptake and phosphorylation of MurNAc-GlcNAc by the PTS transporter MurP. The *SAUSA300_0192* gene was shown to encode a novel 6-phospho-*N*-acetylmuramidase, named MupG that hydrolyses MurNAc 6P-GlcNAc yielding MurNAc 6P and GlcNAc. MupG is the first characterized protein of a so far unexplored family of proteins containing the domain of unknown function 871 (DUF871). Altogether in this study we revealed the recycling pathway of the peptidoglycan sugar turnover product of the Atl autolysin in *S. aureus*.

## Materials and Methods

### Chemicals, Enzymes, and Oligonucleotides

Enzymes for DNA restriction and for cloning were purchased from New England Biolabs (Ipswich, USA) or Thermo Fischer Scientific (Waltham, MA, United States). The Gene JET plasmid miniprep kit, PCR purification kit, and Gene Ruler 1 kb marker were also purchased from Thermo Fisher Scientific and anhydrotetracycline was obtained from Biomol GmbH (Hamburg, Germany). Oligonucleotides were purchased from MWG Eurofins (Ebersberg, Germany) and are listed in Supplementary Table 1.

### Bacterial Strains, Growth Conditions, and Plasmids

The bacterial strains and plasmids used in this study are listed in Supplementary Table 2. The construction of mutant strains and plasmids is also described in the Supplementary Material. *Staphylococcus aureus* USA300 JE2 strain was cultured aerobically in lysogeny broth (LB broth Lennox, Carl Roth) at 37°C and with continuous shaking at 160 rpm or on solid LB supplemented with 1.5% agar. *S. aureus* overnight cultures (∼16 h) were used to inoculate fresh LB medium to yield an initial optical density at 600 nm (OD_600_) of 0.05 for growth studies and for the determination of intracellular accumulation of *N*-acetylmuramic acid 6-phosphate-*N*-acetylglucosamine (MurNAc 6P-GlcNAc) at different growth phases. BM medium (10 g/l peptone, 5 g/l yeast extract, 1 g/l glucose, 5 g/l NaCl, 1 g/l K_2_HPO_4_) was used to prepare electrocompetent *S. aureus* JE2 cells and for the generation of the markerless Δ*mupG* and Δ*mupGmurQ* mutants (see Supplementary Data in the Supplementary Material). Antibiotics were used, when appropriate, at the following concentrations: 100 μg/ml ampicillin and 30 μg/ml kanamycin for *E. coli* and 10 μg/ml chloramphenicol for *S. aureus*.

### Generation of Cytosolic Fractions and Reduction of Samples With NaBH_4_

Overnight cultures of *S. aureus* JE2 wild type, Δ*mupG* and Δ*mupGmurQ* mutants were used to inoculate LB medium to an initial OD_600_ of 0.05. Bacteria were grown at 37°C with constant shaking and harvested at mid-exponential growth phase (OD_600_ of 2.4, grown for ∼3–3.5 h), at transitional phase (OD_600_ of 7.2, grown for 9 h) and stationary phase (OD_600_ of 5.9, grown for 24 h). Afterward, 50 ml of the culture were transferred to Falcon tubes, bacteria were centrifuged at 4,000 × *g* for 10 min, washed with 20 ml deionized water, and pellets were frozen at -80°C. Bacterial samples were thawed at room temperature and suspended in different volumes of water to prepare cell suspensions with OD_600_ of 250 per ml in the different growth phases. Seven hundred microliters of the bacterial suspensions were transferred to new tubes containing ∼0.25 g glass beads (0.25–0.5 mm; Roth) and cells were disrupted in a cell homogenisor (Precellys Evolution, Bertin Technologies) at 6000 rpm for 30 s. This procedure was repeated 4 times, with cooling on ice for 1 min after the second cycle. Lysates were cooled briefly and subsequently centrifuged for 10 min at maximum speed in a microcentrifuge. Two hundred microliters of each supernatant was added to 800 μl of ice-cold acetone to precipitate remaining proteins. After centrifugation (12,000 × *g* for 10 min), the supernatant was transferred to a new tube, and samples were dried under vacuum for 2 h at 55°C and finally stored at 4°C prior to LC-MS measurements.

Reduction solution was freshly prepared for each experiment by adding 500 μl of 0.5 M borate buffer (pH 9) to 5 mg of sodium borohydride, as previously described (Schaub and Dillard, 2017). Thirty microliters of the cytosolic fractions or the culture supernatants were added to equal volumes of the reduction solution and samples were incubated for 20 min at room temperature. Reactions were adjusted to pH between 3 and 4 by adding 10 μl of 8.5% phosphoric acid. Samples were dried under vacuum at 45°C and pellets were solved in 30 μl of deionized water prior to LC-MS analysis.

### Analysis of MurNAc 6P-GlcNAc Accumulation by LC-MS

Sample analysis of bacterial cytosolic fractions was conducted using an electrospray ionization-time of flight (ESI-TOF) mass spectrometer (MicrOTOF II; Bruker Daltonics), operated in negative or positive ion-mode that was connected to an UltiMate 3000 high performance liquid chromatography (HPLC) system (Dionex). For HPLC-MS analysis cytosolic samples were dissolved in 50 μl deionized water and 3 μl were injected into a Gemini C18 column (150 by 4.6 mm, 5 μm, 110 Å, Phenomenex). A 45 min program at a flow rate of 0.2 ml/min was used to separate compounds in the cytosolic fractions as previously described (Gisin et al., 2013). The mass spectra of the investigated samples were presented as base peak chromatograms (BPC), differential spectra (DS, obtained by subtraction of BPC_mutant_ minus BPC_wildtype_) and extracted ion chromatograms (EIC) in DataAnalysis program and were presented in Prism 6 (GraphPad). The relative amounts of MurNAc 6P-GlcNAc in different growth phases and in *mupG* complementation experiments were determined by calculating the area under the curve (AUC) of the respective EIC spectra for MurNAc 6P-GlcNAc.

### Construction of pET28a-*mupG* Plasmid and Heterologous Expression and Purification of MupG-His_6_

For heterologous overexpression in *E. coli*, the *mupG* gene was cloned in a pET28a(+) expression vector as a recombinant protein with a C-terminal His_6_-tag. Therefore, genomic DNA from *S. aureus* USA300 JE2 strain was used to amplify the *mupG* gene by PCR using primer pair MB-67 and MB-68. Both, the PCR product and vector were digested with NcoI and XhoI restriction enzymes, ligated by T4 DNA ligase and chemically competent *E. coli* DH5α cells were transformed with the ligation reaction mixture. The pET28a-*mupG* recombinant plasmid was isolated from kanamycin-resistant *E. coli* cells and DNA sequence was verified by sequencing. BL21(DE3) cells were then transformed with the pET28a-*mupG* plasmid and protein was expressed by addition of IPTG (isopropyl-β-D-thiogalactopyranoside), controlling the T7 inducible promoter.

For expression of MupG, an overnight culture of *E. coli* BL21(DE3) pET28a-*mupG* cells was diluted to an OD_600_ of 0.05 with LB medium supplemented with 30 μg/ml kanamycin (final volume of 1 l) and cells were grown at 37°C with shaking. MupG expression was induced by addition of 1 mM IPTG after the culture reached an OD_600_ of 0.75. Three hours after induction, bacteria were harvested by centrifugation (4,000 × *g*, 4°C, 20 min) and frozen at -20°C. Then, cells were solved in phosphate buffer A (20 mM Na_2_HPO_4_, 500 mM NaCl, pH 8), disrupted by sonication (Branson Sonifier 250, 3 times for 2 min, output 5, duty cycle of 50%) and cell debris were removed by centrifugation (38,000 × *g*, 4°C, 20 min). The soluble fraction was filtered (0.2 μm, Sarstedt) and the His-tagged protein was purified by Ni^2+^ affinity chromatograpy system Äkta Purifier on a 1 ml HisTrap HP column (GE Healthcare). A linear gradient over 30 min from 96% buffer A and 4% buffer B (20 mM Na_2_HPO_4_, 500 mM NaCl, 500 mM imidazole, pH 8) to 100% buffer B was used to elute the His-tagged enzyme from the column. The UV_280nm_ active fractions were analyzed for MupG protein content on 13% SDS-PAGE gel. The MupG-His_6_ containing fractions were pooled and further purified by size-exclusion chromatography (HiLoad 16/600 Superdex 75 pg, GE Healthcare) with buffer A for elution. Protein purity was analyzed by SDS-PAGE and the extinction coefficient at 280 nm of 20,860 M^-1^ cm^-1^ was calculated based on amino acid composition with the ExPASy ProtParam tool. Protein was stored at -80°C in 10% glycerol solution.

### Phylogenetic Analysis

The phylogenetic tree was based on the Pfam entry PF05913. A set of 519 amino acid sequences in this entry (for a complete list of proteins see Supplementary Table 3) were aligned using MAFFT (Katoh and Standley, 2013) under default settings and subsequently trimmed with trimAl in automated1 mode (Capella-Gutiérrez et al., 2009). Pfam PF05913 sequences with less than 200 amino acids were removed from the analysis. The maximum likelihood phylogenetic tree was constructed with RaxML using the Gamma Blosum62 Protein model and rapid bootstrapping algorithm (Stamatakis, 2014). The visualization and annotation was done with the program interactive Tree Of Life (iTOL) v3: an online tool for the display and annotation of phylogenetic and other trees (Letunic and Bork, 2016).

## Results

### Accumulation of MurNAc 6-phosphate-GlcNAc in Δ*mupG* and Δ*mupGmurQ* Cells

**FIGURE 1:**
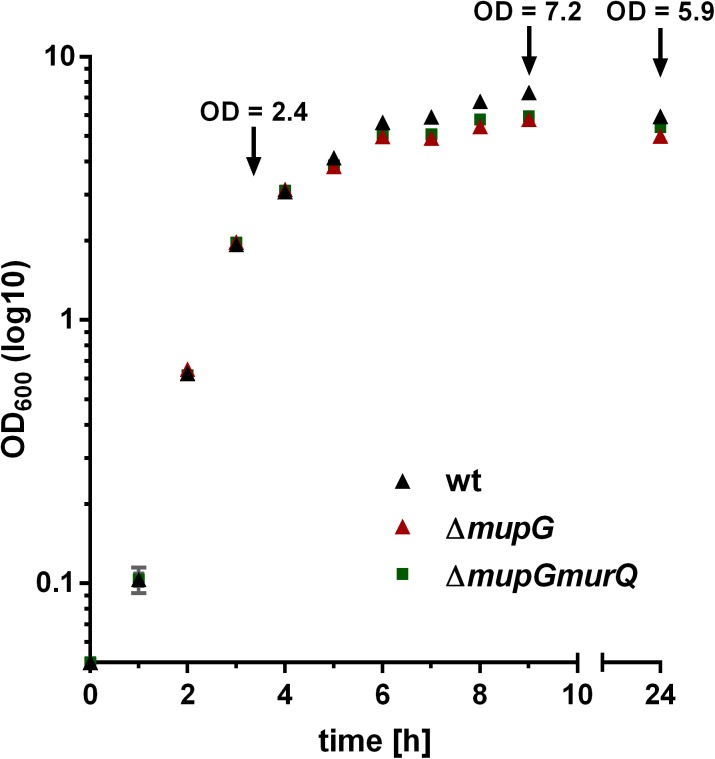
Growth kinetics of wild type and peptidoglycan recycling mutants in LB medium. *S. aureus* wild type (wt, black triangles), Δ*mupG* mutant (red triangle), and Δ*mupGmurQ* (green squares) cells were grown in LB medium under constant shaking and the optical density was monitored at 600 nm (OD_600_) up to 24 h. Experiment was performed in triplicates and data were presented in GraphPad Prism 6 as mean ± SD. Arrows indicate the time when samples were taken for the generation of cytosolic fractions in different growth phases.

The gene *SAUSA300_0192*, named *mupG*, encodes an uncharacterized, hypothetical protein that is classified as a member of a protein family of unknown function (DUF871). It is located upstream of *murQ*, *murP* and *murR*, encoded in an operon on the genome of *S. aureus* strain USA300_FPR3757 (according to the AureoWiki database^1^) (Fuchs et al., 1990). We recently showed that the PTS transporter MurP and the MurNAc 6-phosphate etherase MurQ are required for the recovery of the cell wall sugar *N*-acetylmuramic acid (MurNAc) in *S. aureus* and other Gram-positive bacteria (Borisova et al., 2016). Thus, we assumed that the enzyme MupG of *S. aureus* might also be involved in the recycling of peptidoglycan turnover products, possibly acting sequentially with MurQ within a joint catabolic pathway. To prove this hypothesis, we generated markerless *S. aureus* Δ*mupG* and Δ*mupGmurQ* deletion mutants (see Supplementary Data and Supplementary Figure 1) and we analyzed growth of these mutant strains in comparison with the parental (wild type) strain in nutrient-rich LB medium for 24 h (Figure 1). No significant differences in growth were detectable in all three strains during exponential growth phase, when monitoring the optical density at 600 nm (OD_600_). However, a slight reduction in OD_600_ was observed during late exponential and stationary phase for Δ*mupG* and Δ*mupGmurQ*, compared to the wild type cultures. A *S. aureus* USA300 JE2 Δ*murQ* mutant, lacking the etherase enconding *murQ*, which is located in the same putative operon as *mupG* also showed a slight growth defect in these growth phases, as described previously in Borisova et al. (2016). Therefore, we also compared the growth of the Δ*mupG* mutant with the Δ*murQ* mutant. Interestingly, Δ*mupG* cultures reached slightly lower optical density compared to the Δ*murQ* cultures (see Supplementary Figure 2).

**FIGURE 2:**
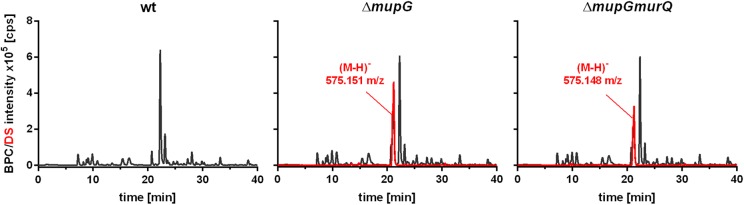
Analysis of cytosolic extracts from wild type, Δ*mupG* and Δ*mupGmurQ* cells by LC-MS. *S. aureus* JE2 wild type (wt) and mutant strains were grown in LB medium for 24 h. Macromolecules were removed from the cytosolic fractions by acetone precipitation and soluble extracts were analyzed by LC-MS in negative ion-mode. Representative mass spectra from at least three independent experiments are presented as base peak chromatograms (BPC) (×10^5^ counts per s [cps]) in black and as differential spectra (DS, BPC_mutant_ – BPC_wt_) (×10^5^ [cps]) in red. DS of both mutants revealed the intracellular accumulation of a compound (absent in the wt cells) with an observed mass of (M-H)^-^ = 575.151 m/z in Δ*mupG* cells and an observed mass of (M-H)^-^ = 575.148 m/z) in Δ*mupGmurQ* cells.

**FIGURE 3:**
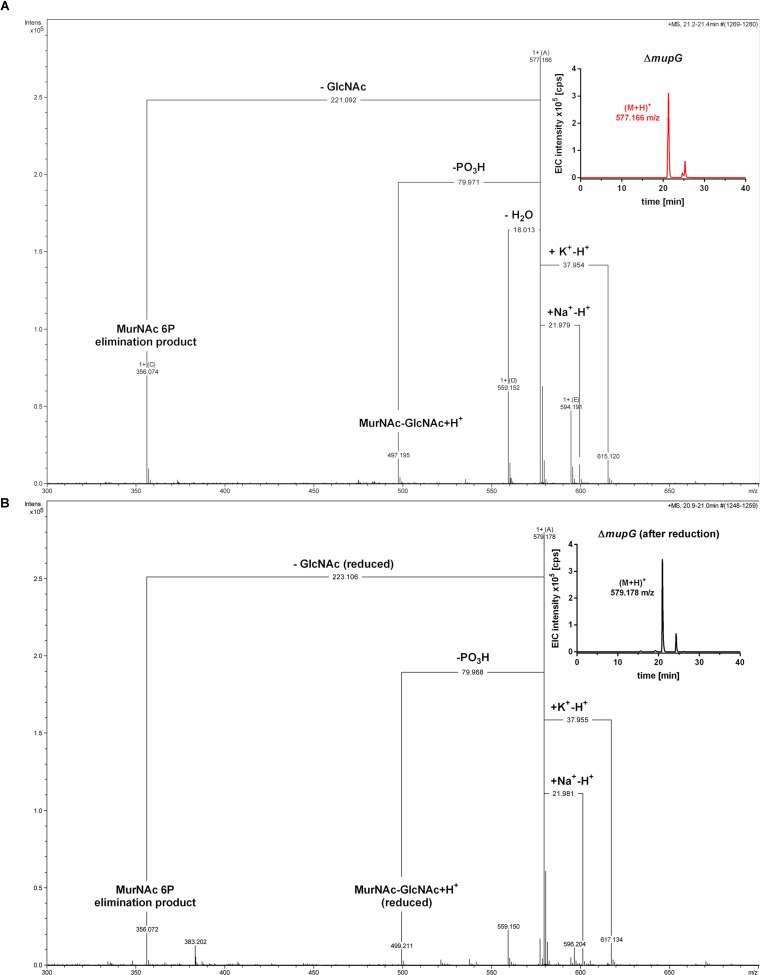
Accumulation of MurNAc 6P-GlcNAc in Δ*mupG* cells. *S. aureus* Δ*mupG* was grown in LB medium to stationary phase (24 h). Acetone extracts of the cytosolic fractions were analyzed by LC-MS in positive ion-mode, before **(A)** and after **(B)** NaBH_4_ reduction. **(A)** Shown are the fragmentation patterns obtained by in-source decay of the compound that accumulates specifically in Δ*mupG* mutant cells and eluates at a retention time of 21.2–21.4 min, represented as extracted ion chromatogram with an observed mass of (M+H)^+^ = 577.166 m/z (EIC × 10^5^ counts per s [cps], upper right corner). Representative mass spectra from three biological replicates are illustrated. **(B)** Shown are the fragmentation patterns after reduction of the sample with NaBH_4_, which results in the formation of a new compound that elutes at 21.0 min, represented by an EIC with an observed mass of (M+H)^+^ = 579.178 m/z (upper right corner). The mass spectra fragmentation patterns of both compounds were presented in Compass DataAnalysis program (Bruker) from 300 to 700 m/z. The obtained fragmentation pattern indicated that the substrate for *MupG* is MurNAc 6P-GlcNAc.

Mass spectrometric analyses of cytosolic cell fractions of Δ*mupG* and Δ*mupGmurQ* cells revealed the accumulation of an intracellular recycling product in both mutant strains, which was absent in wild type cells. The accumulation product in both mutant strains occurred at the same retention time of 21.2 min and had an identical mass within the error of mass analysis of <5 ppm (Δ*mupG* cells, (M-H)^-^ = 575.151 m/z and Δ*mupGmurQ* cells, (M-H)^-^ = 575.148 m/z) (Figure 2). The accumulation of the same compound in both mutants and the absence of MurNAc 6P accumulation in the double mutant, which has been reported to accumulate in a Δ*murQ* single mutant (Borisova et al., 2016), indicated that MupG and MurQ are indeed operating in the same peptidoglycan recovery pathway, with MupG presumably acting upstream of MurQ. The unknown accumulation product has a mass that is identical within the error of mass analysis with the theoretical mass of a phosphorylated disaccharide containing the sugars GlcNAc and MurNAc (calculated mass in negative ion mode (M-H)^-^ = 575.1495 m/z and positive ion mode (M+H)^+^ = 577.164 m/z). To identify the chemical structure of the phosphorylated disaccharide, i.e., which sugar resides at the reducing end and which sugar is phosphorylated, we analyzed the fragmentation patterns obtained by in-source decay during mass analysis. We analyzed the intact cytosolic extracts of the Δ*mupG* cells containing the accumulation product, as well as the same samples after reduction with NaBH_4_ this time in positive ion-mode (Figure 3). As expected a mass shift of 2 Da occurred after treatment with NaBH_4_, in agreement with the expected reduction of a sugar hemiacetal at the reducing end yielding a sugar alcohol. The accumulation product in the Δ*mupG* extract with a mass of (M+H)^+^ = 577.166 m/z (non-reduced) was diminished [Figure 3A; calculated mass of a phosphorylated disaccharide containing GlcNAc and MurNAc in positive ion mode: (M+H)^+^ = 577.164 m/z)], and a new mass appeared with (M+H)^+^ = 579.178 m/z, corresponding to the same disaccharide reduced to sugar alcohol [calculated reduced form (M+H)^+^ = 579.1797 m/z] (Figure 3B). The fact that the phosphorylated disaccharide is reduced at the anomeric position indicated that the C1 position is not phosphorylated, but most likely the C6 position carries the phosphate group. Thus, four different chemical structures are imaginable, which were presented in Supplementary Figure 3A.

In the non-reduced sample (Figure 3A), the recycling product with the mass of (M+H)^+^ = 577.166 m/z was detected and also the Na^+^ and K^+^ adducts. Fragmentations included the neutral loss of GlcNAc (-221.092 Da; calculated exact mass loss of 221.089 Da), and a MurNAc 6P elimination product (see Supplementary Figure 3B) with a mass of (M+H)^+^ = 356.074 m/z (which is identical with the calculated m/z of this fragment). Besides, the loss of a phosphoryl group (-79.971 Da) and the loss of water (-18.013 Da), most likely at the anomeric site, was observed. The latter fragmentations caused the formation of a dephosphorylated recycling product [(M+H)^+^ = 497.195 m/z] and a dehydrated product [(M+H)^+^ = 559.152 m/z], respectively. From these MS result we could conclude that MurNAc, but not GlcNAc, is phosphorylated. After sample reduction, the mass of the accumulation product changed to (M+H)^+^ = 579.178 m/z (Figure 3B). Na^+^ and K^+^ adducts of the reduced compound were also detected. Compound fragmentation pattern included the neutral loss of GlcNAc, this time in a reduced form (measured and theoretical exact mass loss of 223.106 Da) and generated a compound, as seen in the non-reduced sample, with a m/z identical within error with a MurNAc 6P elimination product (356.072 m/z) (Figure 3B; see also Supplementary Figure 3B). Fragments indicating the loss of phosphate (-79.968 Da, resulting in 499.211 m/z, reduced MurNAc-GlcNAc) were detected also in the reduced sample. However, a fragmentation product indicating the loss of water was absent, as expected for a compound reduced at the anomeric position. These latter results showed that after sample reduction with NaBH_4_, the mass of the elimination product of MurNAc 6P did not change, but the mass, corresponding to GlcNAc, was changed to the reduced form. Thus, we could conclude that the investigated phosphorylated disaccharide contains a MurNAc at the non-reducing end that is phosphorylated and GlcNAc at the reducing end. Taken together, the fragmentation patterns of the non-reduced and reduced accumulation products, identified the accumulation of MurNAc 6P-GlcNAc disaccharide in the Δ*mupG* cells.

### MurNAc 6P-GlcNAc Is Hydrolyzed by MupG Yielding MurNAc 6P and GlcNAc

**FIGURE 4:**
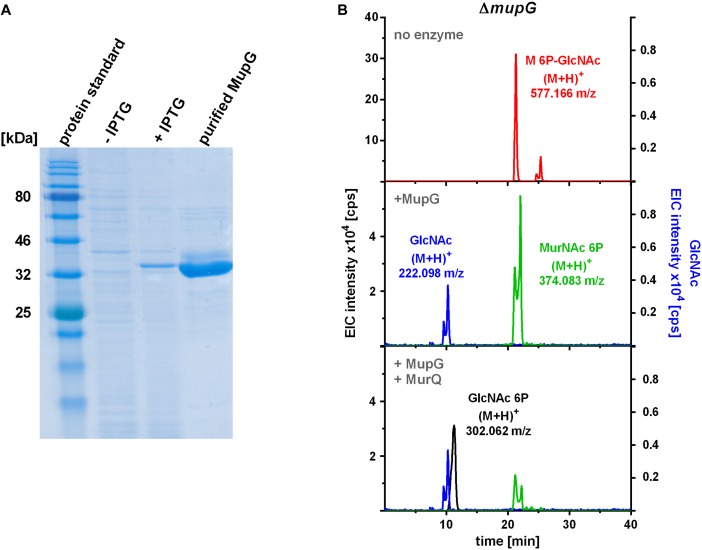
Production of recombinant MupG and enzymatic digest of MurNAc 6P-GlcNAc extracted from Δ*mupG* cytosolic fractions. **(A)** MupG from *S. aureus* JE2 strain was heterologously expressed in *E. coli* BL21(DE3) cells. Protein expression, before (-IPTG) and after induction for 3 h (+IPTG), and purity (after purification by Ni^2+^-affinity and size exclusion chromatography) was controlled by SDS-PAGE (5 μg of the recombinant MupG protein was loaded). MupG-His_6_ protein has a calculated molecular weight of 41.5 kDa. **(B)** *S. aureus* Δ*mupG* cells were grown in LB medium for 24 h. Acetone extracts of the cytosolic fractions were analyzed by LC-MS in positive ion-mode. Fifty microliter of the cytosolic fraction from Δ*mupG* was incubated with buffer (no enzyme), with 1 μg recombinant MupG (+MupG) or with MupG and MurNAc 6P etherase MurQ (both 1 μg) for 2.5 h at 37°C. Representative mass spectra of the samples from at least three biological replicates were presented as extracted ion chromatograms (EIC × 10^4^ [cps]) for MurNAc 6P-GlcNAc (observed masses of (M+H)^+^ = 577.166 m/z) in red, GlcNAc ((M+H)^+^ = 222.098 m/z) in blue, MurNAc 6P ((M+H)^+^ = 374.083 m/z) in green and GlcNAc 6P ((M+H)^+^ = 302.062) in black.

To characterize the *in vitro* functionality of MupG from *S. aureus*, we overexpressed the enzyme in *E. coli* BL21(DE3) in the presence of IPTG as a recombinant C-terminal His_6_-fusion protein (Figure 4A) and purified the recombinant enzyme by Ni^2+^-affinity and gel filtration chromatography (calculated mass of 41.5 kDa). After purification, a total amount of 3 and 5 mg protein, respectively, was obtained from 1 l bacterial culture in two different experiments and purity of the purified enzyme was confirmed with SDS-PAGE (Figure 4A). Addition of the recombinant MupG enzyme to cytosolic extracts, prepared from wild type and Δ*mupG* mutant cells revealed the specific cleavage of the Δ*mupG* recycling product ((M+H)^+^ = 577.166 m/z, measured in positive ion-mode, and a retention time of 21.2 min), yielding two new masses (M+H)^+^ = 222.098 m/z and (M+H)^+^ = 374.083 m/z (Figure 4B). These new masses correspond to the theoretical masses for GlcNAc [(M+H)^+^ = 222.097 m/z] and MurNAc P [(M+H)^+^ = 374.085 m/z], respectively. However, MupG did not affect any other compound accumulating in the cytosol of the wild type cells or Δ*mupG* cells (data not shown). Notably, in extracts of the mutant, we detected a second peak with a mass of (M+H)^+^ = 577.165 m/z that eluted with a retention time of 25 min (Figure 4B). This compound was only detectable, when the MS analysis was conducted in the positive ion-mode, but was absent when samples were analyzed in negative ion-mode (see Figures 1, 2 for comparison). Recombinant MupG enzyme only diminished the compound with a retention time of 21.2 min but not the compound with the same m/z but a retention time of 25 min (Figure 4B). The identity of this compound is so far unclear, since the MS fragmentation pattern indicated that it is neither composed of non-phosphorylated nor of phosphorylated MurNAc and GlcNAc sugars (data not shown).

To unequivocally identify MurNAc 6P as the product of MupG cleavage, we incubated the MupG-treated cytosolic fraction, with the MurNAc 6P etherase MurQ, which specifically converts MurNAc 6P to GlcNAc 6P (Borisova et al., 2016). As expected, MurQ reduced the amount of MurNAc 6P about 4 times, at the same time a mass with (M+H)^+^ = 302.062 m/z, corresponding to the theoretical mass of GlcNAc 6P ((M+H)^+^ = 302.064 m/z) was generated (Figure 4B). MurQ treatment did not affect amounts of GlcNAc, the second product of MupG action. These results confirmed that the accumulating recycling product is indeed MurNAc 6P-GlcNAc and that the MupG enzyme acts as a MurNAc 6P-GlcNAc glycosidase. The recombinant enzyme was stable for several months at 4°C without losing significant activity, even in the absence of DTT reducing agent.

### MurNAc 6P-GlcNAc Accumulation Primarily Occurs in Transition and Stationary Phase and Is Abolished by Plasmid-Based Complementation of MupG

We previously showed that a *murQ* mutant of *S. aureus* accumulates intracellularly MurNAc 6P predominantly in transition and stationary phases (Borisova et al., 2016). Here, we examined the growth phase-dependent accumulation of MurNAc 6P-GlcNAc. The time points used for harvesting where mid exponential growth phase (3 h), early stationary phase (9 h) and stationary phase (24 h). These growth phases were defined by a growth kinetics study with *S. aureus* wild type and Δ*mupG* mutant cells in LB medium (cf. Figure 1). The accumulation of MurNAc 6P-GlcNAc was low at exponential phase, increased significantly at transition phase and reached the highest level after 24 h of growth (Supplementary Figure 4). Thus, accumulation of MurNAc 6P-GlcNAc in Δ*mupG* cells confirmed that cell wall sugar recycling occurs predominantly in the transition and stationary phase.

**FIGURE 5:**
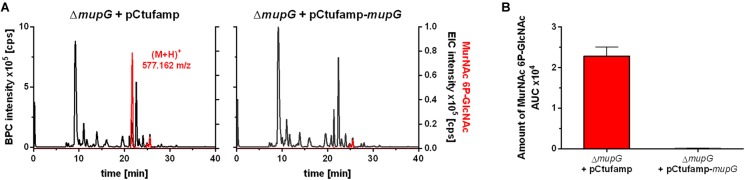
LC-MS analysis of cell extract from MupG complemented mutant. *S. aureus* Δ*mupG* cells transformed with the control pCtufamp plasmid (Δ*mupG* + pCtufamp) or with the *mupG* complementation plasmid pCtufamp-*mupG* (Δ*mupG* + pCtufamp-*mupG*) were grown for 24 h. Cytosolic fractions were analyzed by LC-MS in positive ion-mode. **(A)** Mass spectra of the samples were presented as base peak chromatograms (BPC × 10^5^ counts per s [cps]) in black and as extracted ion chromatograms (EIC × 10^5^ [cps]) for MurNAc 6P-GlcNAc with observed mass of (M+H)^+^ = 577.162 m/z in red. **(B)** MupG complementation experiment was performed in triplicates and the relative amount of the MurNAc 6P-GlcNAc in the cells was determined by calculating the area under the curve (AUC) of the respective EIC spectra.

We further investigated, whether accumulation of MurNAc 6P-GlcNAc in the Δ*mupG* cells can be abolished by expressing MupG on a plasmid. We used stationary phase cells for this study, since highest levels of accumulation were obtained at this stage (Supplementary Figure 4). *S. aureus* Δ*mupG* cells were transformed with a plasmid (named pCtufamp-*mupG;* see Supplementary Data) constitutively expressing MupG, or with empty plasmid (pCtufamp) as a control. Analysis of the cytosolic fractions of the Δ*mupG* cells showed that MupG expression leads to complete disappearance of MurNAc 6P-GlcNAc amounts in the cytosol after 24 h of growth (Figure 5).

### MurNAc-GlcNAc Accumulates Extracellularly in *S. aureus* Cells Lacking the MurP Transporter

**FIGURE 6:**
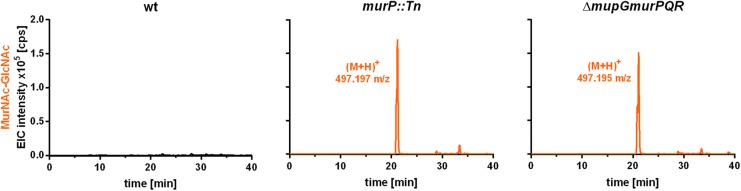
LC-MS analysis from recycling mutants, lacking MurP transporter. *S. aureus* wild type (wt) cells, a *murP* transporter mutant (*murP::Tn*) and a *murQ*-operon mutant (Δ*mupGmurQPR*) were grown to stationary phase (24 h) in LB. Bacterial cultures were centrifuged and 1 ml of each supernatant was transferred to 500 μl of ice-cold acetone to remove macromolecules and proteins from the samples. After centrifugation, culture supernatants were dryed in the speedvac, solved in 50 μl of Millipore water and 2 μl of the samples were injected to the HPLC column. MS analysis of the culture supernatants was performed in positive-ion mode and mass spectra are presented as extracted ion chromatograms (EIC × 10^5^ counts per second [cps]) in orange. In the culture supernatants of both mutants, an additional compound accumulates (*murP::Tn*, observed mass of (M+H)^+^ = 497.197 m/z; Δ*mupGmurQPR*, observed mass of (M+H)^+^ = 497.195 m/z). The mass of this compound in the supernatant corresponds to a disaccharide made of GlcNAc and MurNAc. This compound was further analyzed by MS fragmentation and identified as MurNAc-GlcNAc (see Supplementary Figure 5). MS data are shown as representative from three biological replicates.

The PTS transporter MurP of *S. aureus* was shown to transport and concomitantly phosphorylate MurNAc, yielding MurNAc 6P (Borisova et al., 2016). However, MurNAc-GlcNAc rather than MurNAc is the main sugar released from the peptidoglycan by Atl and other autolysins during cell wall turnover in *S. aureus.* Thus, we proposed that MurNAc-GlcNAc might be primary the natural substrates of MurP, thereby generating intracellular MurNAc 6P-GlcNAc, the substrate of the MupG hydrolase. To test this hypothesis, we analyzed the extracellular extracts of *S. aureus* wild type and two *murP* mutant cells (*murP::Tn* insertion mutant and Δ*mupGmurQPR* markerless deletion mutant). The MS analysis of the culture supernatant indeed revealed the accumulation of a disaccharide turnover product with a mass identical to MurNAc-GlcNAc (observed (M+H)^+^ = 497.197 and 497.195 m/z, respectively, theoretical mass of (M+H)^+^ = 497.198 m/z), which was lacking in the culture supernatant of the wild type cells (Figure 6). To confirm the identity of the disaccharide, again MS fragmentation analyses before and after reduction were conducted, as summarized in Supplementary Figure 5. A compound with the mass of (M+H)^+^ = 497.197 m/z was detected also as Na^+^ and K^+^ adducts, as well as it was characterized by the neutral loss of GlcNAc (-221.087 Da) and the formation of a MurNAc elimination product [(M+H)^+^ = 276.111 m/z]. In addition, we identified a fragmentation product in which water was eliminated (-18.004 Da) yielding a dehydration product [(M+H)^+^ = 479.192 m/z] (Supplementary Figure 5A). After NaBH_4_ reduction of the culture supernatant from the *murP::Tn* mutant, the mass of the disaccharide product (M+H)^+^ = 497.197 m/z disappeared and a product in a reduced form with a mass of (M+H)^+^ = 499.214 m/z appeared. This product was also detected as Na^+^ and K^+^ adducts and, in addition, the fragmentation pattern revealed a mass corresponding to a MurNAc elimination product (observed mass of 276.108 m/z) that lost a neutral mass, corresponding to the exact mass of reduced GlcNAc (-223.107 Da). The absence of a fragmentation product with a loss of water is in agreement with a reduction at the anomeric site, which precludes water elimination. The fragmentation pattern of the non-reduced and reduced extracellular accumulation products indicated that MurNAc-GlcNAc, but not GlcNAc-MurNAc, accumulates in the culture supernantant of Δ*murP* deletion mutants. This disaccharide was not cleaved by the recombinant β-1,4-*N*-acetylglucosaminidase NagZ from *B. subtilis* (Litzinger et al., 2010a,b) (data not shown), which provides a further evidence that GlcNAc-MurNAc is not the recycling product in *S. aureus*. The same MS fragmentation pattern was obtained in the culture supernantant of the Δ*mupGmurQPR* recycling mutant, before and after reduction of the supernatant (data not shown), showing that also in this mutant the recycling product MurNAc-GlcNAc accumulates. Interestingly, in the culture supernatant of the Δ*mupGmurQPR* mutant also other compounds accumulated and were absent in the supernatant of the wild type cells, which nature could not be identified so far (data not shown).

To conclude, the extracellular accumulation of MurNAc-GlcNAc in both *murP* mutants, as well as the intracellular accumulation of MurNAc 6P-GlcNAc in the *mupG* mutants and the absence of MurNAc 6P-GlcNAc and MurNAc 6P in the cytosol of Δ*mupGmurQPR* operon mutant revealed that the disaccharide MurNAc-GlcNAc is the natural substrate internalized by the PTS transporter MurP, which intracellularly yields MurNAc 6P-GlcNAc, the substrate of MupG.

## Discussion

**FIGURE 7:**
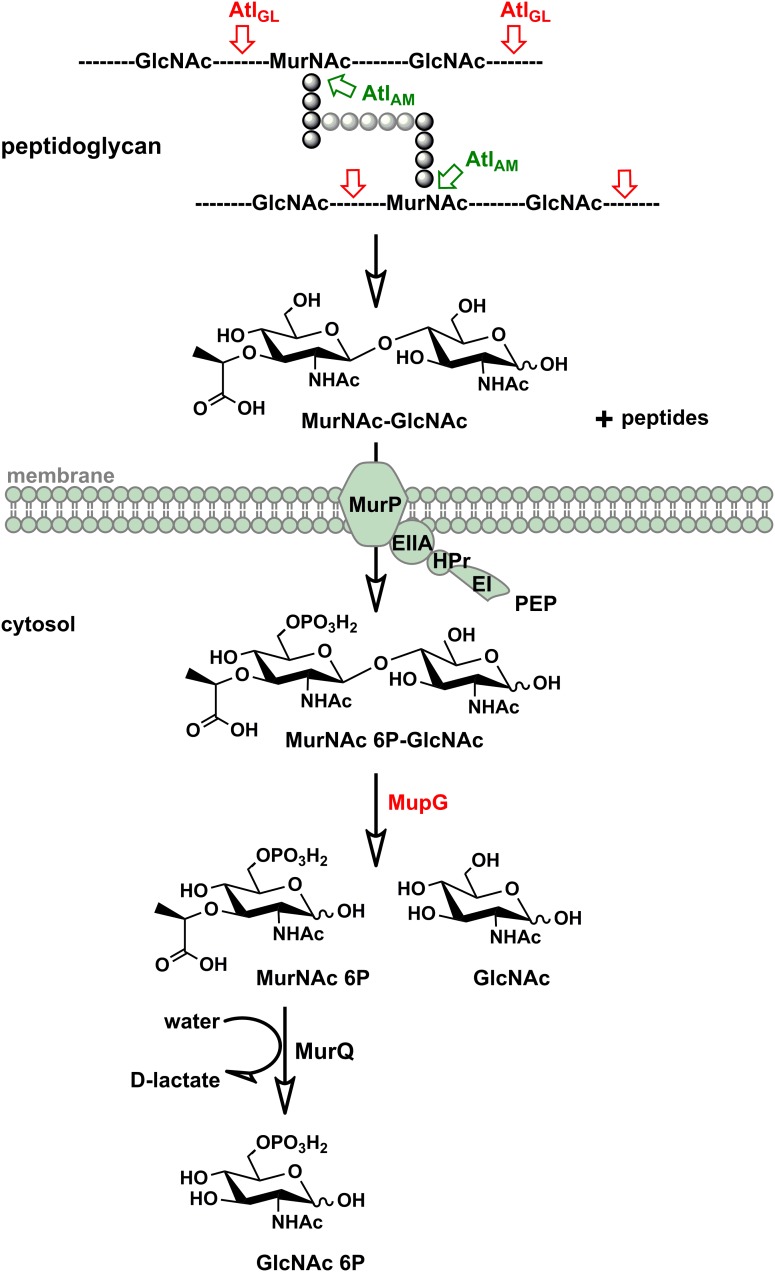
Scheme of the peptidoglycan sugar recycling in *S. aureus*. During growth and division, *S. aureus* cells constantly degrade and resynthesize their peptidoglycan. The major autolysin Atl, a bifunctional muramoyl-L-alanine amidase and endo-*N*-acetylglucosaminidase, is able to cleave its peptidoglycan, generating MurNAc-GlcNAc and peptide turnover products. MurNAc-GlcNAc disaccharide is reutilized: transported in the cells and concommitantly phosphorylated by the MurP PTS transporter generating intracellularly MurNAc 6P-GlcNAc. Subsequently, the MurNAc 6-phosphate-GlcNAc glycosidase MupG cleaves this compound, generating the products MurNAc 6P and GlcNAc. The D-lactyl ether substituent of MurNAc 6P is specifically cleaved off by the etherase MurQ, previously characterized in our lab, forming GlcNAc 6P and D-lactate (Borisova et al., 2016). It is currently unclear how GlcNAc, the second product of the MupG reaction, is further metabolized in *S. aureus* cells.

We discovered in this study how *S. aureus* reutilizes the sugar part of the peptidoglycan of its cell wall. The overall scheme of this novel peptidoglycan recycling pathway is depicted in Figure 7. Mass spectrometric analysis of sugars compounds, accumulating in the growth medium of Δ*murP* cells (MurNAc-GlcNAc) and in the cytoplasm of Δ*mupG* cells (MurNAc 6P-GlcNAc), measured directly or after reduction of the sugars, unequivocally demonstrated the chemical nature of these compounds and allowed to establish the pathway. Thus, the principle peptidoglycan turnover product of *S. aureus* is MurNAc-GlcNAc, which results from the peptidoglycan cleavage by muramoyl-L-Ala amidases and endo-*N*-acetylglucosaminidases, whereas lysozyme-like endo-*N*-acetylmuramidases would generate GlcNAc-MurNAc products. Remarkably, the peptidoglycan of *S. aureus* is frequently *O*-acetylated at the C6 hydroxyl group of MurNAc, thereby rendered lysozyme-resistant (Bera et al., 2006; Sychantha et al., 2017). An endogenous lysozyme-like muramidase therefore would not be able to cleave the peptidoglycan of *S. aureus*. Accordingly, *S. aureus* possesses peptidoglycan-cleaving endo-*N*-acetylglucosamindases: one of them is the well-studied major autolysin Atl (Sugai et al., 1995; Wheeler et al., 2015; Chan et al., 2016).

Since only MurNAc-GlcNAc and not MurNAc was found in the spent medium of the *murP::Tn* and Δ*mupGmurQPR* mutants during growth, the disaccharide appears to be the general peptidoglycan turnover product of *S. aureus* and natural substrate of the PTS transporter MurP. However, MurP had been shown previously to take up and phosphorylate also MurNAc, if added to the growth medium (Borisova et al., 2016). Thus, apparently MurP accepts both sugars, MurNAc and MurNAc-GlcNAc as substrates. To our knowledge, this is the first report of a PTS system that is able to take up and to specifically phosphorylate a disaccharide as well as a monosaccharide (Postma et al., 1993). Since *murP* of *S. aureus* encodes only the enzyme IIBC components, an enzyme IIA (along with the general components EI and HPr of the PTS) is required to enable MurP functioning (Figure 7). So far, it is unclear which enzyme IIA operates together with MurP.

The most important result of this study is the identification of a novel 6-phosphomuramidase, encoded by *SAUSA300_0192* (strain USA300), which we named MupG, for MurNAc-6P glycosidase. MurNAc 6P-GlcNAc accumulated in Δ*mupG* cells of *S. aureus* and recombinantly produced MupG cleaved MurNAc 6P-GlcNAc, releasing MurNAc 6P and GlcNAc. The former product is subsequently metabolized and was identified by specific cleavage by the MurNAc 6P etherase MurQ, yielding GlcNAc 6P and D-lactate (Borisova et al., 2016). Moreover, accumulation of MurNAc 6P-GlcNAc in a Δ*mupG* mutant was abolished by complementation using a MupG-expressing plasmid. Since only accumulation of MurNAc 6P-GlcNAc and not of MurNAc 6P was observed in a Δ*murGmurQ* double mutant (please note that MurNAc 6P accumulates in Δ*murQ* cells; Borisova et al., 2016), it can be concluded that recycling of the sugar part of the peptidoglycan during growth of *S. aureus* in LB medium exclusively proceeds via the uptake of MurNAc-GlcNAc disaccharides.

6-phosphoglycosidases commonly act in combination with PTS glycoside transporters. Such systems were characterized with specificities for α-glucosides such as maltose, sucrose, and trehalose (EC 3.2.1.122), or for β-glycosides (EC 3.2.1.86) or β-galactosides (EC 3.2.1.85), such as cellobiose, chitobiose, and lactose (Hengstenberg et al., 1993; Mokhtari et al., 2013). For example, *S. aureus* (as well as *Lactobacillus* and *Streptococcus* sp.) imports lactose via the specific PTS LacE (enzyme IIBC), which phosphorylates the disaccharide at the C6 hydroxyl group of the β-galactose moiety (Hengstenberg et al., 1993). In addition, it possesses a cytoplasmic 6-phospho-β-galactosidase (LacG) that hydrolyzes lactose 6P to galactose 6P and glucose (Staedtler et al., 1995; Yip et al., 2007; Hill and Reilly, 2008). All of the 6P-glycosidases characterized to date are classified within the CAZy glycosidase families 1 and 4 (Davies et al., 2005), which operate by very distinct mechanisms (Witt et al., 1993; Staedtler et al., 1995; Yip et al., 2007; Hill and Reilly, 2008). MupG, however, displays no significant similarity with these glycosidases, instead it founds an entirely new enzyme family. MupG and related proteins so far had been classified as domain of unknown function proteins (IPR008589; DUF871). Although they had been grouped within the glycoside hydrolase (IPR017853) and aldolase-type TIM barrel (IPR013785) superfamilies, no clear attribution to glycosidase function had been made by bioinformatic analyses. The mechanism of members of this novel glycosidase family, i.e., the stereochemical outcome and catalytic residues remain enigmatic. Our results, however, indicate that MupG has no requirement for NAD^+^ as for family 4 glycosidases (Yip et al., 2007).

**FIGURE 8:**
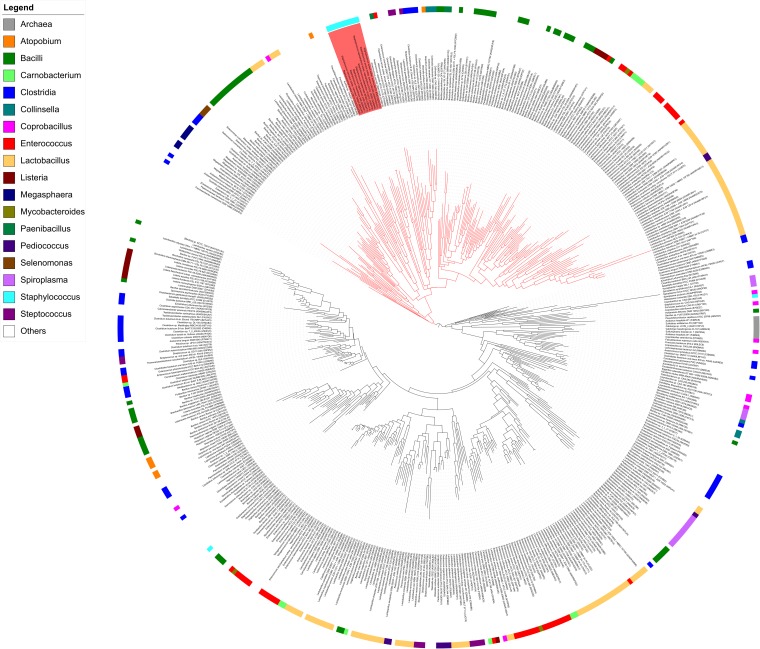
Phylogenetic tree showing the relation of MupG orthologs. The phylogenetic tree was constructed with RaxML from an alignment of 519 amino acid sequences, based on the Pfam entry PF05913, which lists putative orthologs of MupG (DUF871 domain proteins). The genus of *Staphylococcus* is marked by a red rectangle and includes MupG of *S. aureus* USA300, which was characterized in this study as a MurNAc 6P-glycosidase. Putative MurNAc 6P glycosidase (close orthologs of MupG) constitute a distinct clade, indicated by red lines. More distantly related MupG-like proteins constitute different clades that are indicated by black lines. Bacterial genera are color coded as indicated. See Supplementary Table 3 for a complete list of the proteins within this phylogenetic tree.

Proteins displaying significant amino acid sequence identities with MupG of *S. aureus* can be found in different bacterial species. Together with MupG these proteins constitute a family (Pfam PF05913) containing domain of unknown function (DUF) 871, with MupG representing the first characterized enzyme of this protein family. The distribution of putative orthologous proteins of MupG is surprisingly narrow and mostly restricted to the phylum firmicutes. The reason for this narrow phylogenetic distribution among the firmicutes and a very selective occurrence within organisms of other phyla, is currently unclear. It may suggest a recent evolutionary event and in addition the distribution by horizontal gene transfer, however, additional studies are required to confirm these assumptions. To learn more about the distribution of MupG orthologs within bacteria, we constructed a phylogenetic tree, based on an alignment of 519 amino acid sequences extracted from the protein family entry Pfam PF05913 (Figure 8) that includes proteins from 115 bacterial genera as well as from five Crenarcheotae and one nematode species (see Supplementary Table 3 for a complete list of species or genera containing putative enzymes orthologous to MupG). Intriguingly, the phylogenetic tree revealed distinct clades of proteins that include close homologs of MupG and more distinct MupG-like proteins (Figure 8). Potential orthologs of MupG of *S. aureus* are found in various *Staphylococcus* sp. (red rectangle, Figure 8) and in many other Bacilli, e.g., Bacillales such as *Listeria, Paenibacillus, and Bacillus sp.*, and also in Lactobacillales, e.g., *Streptococcus*, *Lactococcus*, *Enterococcus*, *Lactobacillus*, and *Pediococcus* sp., as well as in some Clostridiales. Intriguingly, also some few Fusobacteriales, Chlamydiales and Spirochetales species as well the nematode *Trichuris trichiura* possess a putative MupG ortholog. Some bacterial species, however, contain up to six putative paralogs of MupG (Supplementary Table 3), indicating that the PF05913/DUF871 protein family besides MupG orthologs might also contain proteins of distinct physiological function and with altered substrate specificity. For example, *Coprobacillus cateniformis* contains six putative paralogs, *Carnobacterium maltaromaticum* LMA28 five, *Lactobacillus plantarum* WCFS1 four, *Bacillus anthracis* and *Enterococcus faecalis* V583 three and *Lactococcus lactis* IL1403, *Bacillus megaterium* and many other bacteria contain two putative paralogs (cf. Supplementary Table 3). We assume that close homologs of MupG of *S. aureus* have identical function (MurNAc-6P glycosidases, see protein clade colored red in Figure 8) but more remotely related proteins (colored black in Figure 8) likely contain enzymes of different function. Remarkebly, some archaea (e.g., *Sulfolobus solfatorius* and *Sulfurisphaera tokodaii*) contain putative MupG-like orthologs. Since archaea lack a peptidoglycan cell wall it seems likely that these enzymes have a function that differs from the hydrolysis of a peptidoglycan turnover product. Many firmicutes presumably contain a MupG ortholog and supposably possess a recycling pathway for MurNAc-GlcNAc as that presented here for *S. aureus*. Notably, crystal structures of two putative orthologs of *MupG* [from *Bacillus cereus* (1×7f) and *Enterococcus faecalis* (2P0O)] were deposited to the protein structure database^2^. We are currently investigating whether these enzymes and MupG have the same specificity and function.

For a long time, the need for cell wall recycling in Gram-positive bacteria, and particularly in coccoidal bacteria such as *S. aureus*, had been questioned. Although, reports from the 1970s revealed a massive turnover of the cell wall in *S. aureus* (Wong et al., 1974; Blümel et al., 1979; reviewed in Doyle et al., 1988; Reith and Mayer, 2011). Recent studies, showed that the peptidoglycan of the cell wall in *S. aureus* undergoes a massive rearrangement during growth and division that involves glycan strand trimming by endo-*N*-acetylglucosaminidases (Wheeler et al., 2015; Chan et al., 2016), peptidoglycan degradation by autolysins such as Atl during cell division and separation (Yamada et al., 1996; Biswas, 2009; Götz et al., 2014) as well as autolysis during biofilm formation (Bose et al., 2012). We recently demonstrated that peptidoglycan recycling proceeds in Gram-positive organisms predominantly occurring during transition and stationary phases (Borisova et al., 2016). The results of the present study confirmed these previous observations. Notably, the *mupG* mutant and the Δ*mupGmurQ* double mutant showed a weak disadvantage during growth in stationary phase (cf. Supplementary Figure 2). This growth defect was stronger than previously observed for the Δ*murQ* mutant (Borisova et al., 2016), which might be explained by the inability of the former mutants to recycle both sugars of the peptidoglycan, GlcNAc and MurNAc, whereas the latter mutant has a defect only in the recovery of MurNAc. So far, it remains unclear how the GlcNAc part of MurNAc 6P-GlcNAc is recovered, after cleavage by MupG in the cytoplasm. GlcNAc reutilization would require either a GlcNAc kinase, or alternatively the secretion and a re-import by the GlcNAc PTS of *S. aureus*, in both cases yielding GlcNAc 6P, which can be shuttled into the amino sugar catabolic pathway (Komatsuzawa et al., 2004). Moreover, the fate of the peptide part of the peptidoglycan is currently unknown.

## Conclusion

Here we elucidated a novel pathway for the uptake and catabolism of the disaccharide MurNAc-GlcNAc, which is a specific peptidoglycan turnover product generated by the joint action of peptidoglycan amidases and endo-*N*-acetylglucosamidases, e.g., by the bifunctional autolysin Atl of *S. aureus*. The pathway in *S. aureus* involves the PTS transporter MurP, required for the uptake and phosphorylation of the disaccharide yielding MurNAc 6-phosphate-GlcNAc, and MupG, a unique glycosidase with 6-phospho-*N*-acetylmuramidase activity. MupG is the first characterized representative of a so far unexplored protein family containing domain of unknown function DUF871, which is distributed mostly among firmicutes. As many of these organisms also possess peptidoglycan-cleaving endo-*N*-acetylglucosaminidases and MurP-like transporters, besides MupG orthologs, recycling of the peptidoglycan turnover product MurNAc-GlcNAc is presumably a common feature among these firmicutes. Peptidoglycan recycling is crucial for stationary phase survival of Gram-positive bacteria (Borisova et al., 2016) and thus, the pathway identified in this study may serve as a novel target to treat persistent infections by *S. aureus* and selectively other bacteria.

## Author Contributions

RK, MB, and PE cloned MupG. RK and MB expressed and biochemically characterized MupG. RK and MB performed HPLC-MS experiments and analyzed the data. MA, RK, and NZ aligned the MupG-like protein family, constructed the phylogenetic tree based on this alignment, and prepared the Figure 8. MB and CM formulated the original problem and provided guidance throughout the study. MB, CM, RK, and PE designed the experiments and developed the methodology. MB, CM, and RK wrote the manuscript. MB approved the final version to be published.

## Conflict of Interest Statement

The authors declare that the research was conducted in the absence of any commercial or financial relationships that could be construed as a potential conflict of interest.

## Footnotes

1 http://aureowiki.med.uni-greifswald.de/SAUSA300_0192

2 https://www.rcsb.org/

## References

[B1] Bera A. Biswas R. Herbert S. Götz F. (2006). The presence of peptidoglycan O-acetyltransferase in various staphylococcal species correlates with lysozyme resistance and pathogenicity. *Infect. Immun.* 74 4598–4604. 10.1128/IAI.00301-06 16861647 PMC1539615

[B2] Biswas R. (2009). Characterization of *Staphylococcus aureus* peptidoglycan hydrolases and isolation of defined peptidoglycan structures. Ph.D. thesis, University of Tübingen, Tübingen.

[B3] Biswas R. Voggu L. Simon U. K. Hentschel P. Thumm G. Götz F. (2006). Activity of the major staphylococcal autolysin Atl. *FEMS Microbiol. Lett.* 259 260–268. 10.1111/j.1574-6968.2006.00281.x 16734789

[B4] Blümel P. Uecker W. Giesbrecht P. (1979). Zero order kinetics of cell wall turnover in *Staphylococcus aureus*. *Arch. Microbiol.* 121 103–110. 10.1007/BF00689972 485764

[B5] Boneca I. G. Huang Z. H. Gage D. A. Tomasz A. (2000). Characterization of *Staphylococcus aureus* cell wall glycan strands, evidence for a new β-N-acetylglucosaminidase activity. *J. Biol. Chem.* 275 9910–9918. 10.1074/jbc.275.14.9910 10744664

[B6] Borisova M. Gaupp R. Duckworth A. Schneider A. Dalügge D. Mühleck M. (2016). Peptidoglycan recycling in Gram-positive bacteria is crucial for survival in stationary phase. *mBio* 7:e00923–16. 10.1128/mBio.00923-16 27729505 PMC5061867

[B7] Bose J. L. Lehman M. K. Fey P. D. Bayles K. W. (2012). Contribution of the *Staphylococcus aureus* Atl AM and GL murein hydrolase activities in cell division, autolysis, and biofilm formation. *PLoS One* 7:e42244. 10.1371/journal.pone.0042244 22860095 PMC3409170

[B8] Cabeen M. T. Jacobs-Wagner C. (2005). Bacterial cell shape. *Nat. Rev. Microbiol.* 3 601–610. 10.1038/nrmicro1205 16012516

[B9] Capella-Gutiérrez S. Silla-Martínez J. M. Gabaldón T. (2009). trimAl: a tool for automated alignment trimming in large-scale phylogenetic analyses. *Bioinformatics* 25 1972–1973. 10.1093/bioinformatics/btp348 19505945 PMC2712344

[B10] Chan Y. G. Frankel M. B. Missiakas D. Schneewind O. (2016). SagB glucosaminidase is a determinant of *Staphylococcus aureus* glycan chain length, antibiotic susceptibility, and protein secretion. *J. Bacteriol.* 198 1123–1136. 10.1128/JB.00983-15 26811319 PMC4800868

[B11] Cho H. Wivagg C. N. Kapoor M. Barry Z. Rohs P. D. Suh H. (2016). Bacterial cell wall biogenesis is mediated by Seds PBP polymerase families functioning semi-autonomously. *Nat. Microbiol.* 1:16172 10.1038/nmicrobiol.2016.172 27643381 PMC5030067

[B12] Davies G. J. Gloster T. M. Henrissat B. (2005). Recent structural insights into the expanding world of carbohydrate-active enzymes. *Curr. Opin. Struct. Biol.* 15 637–645. 10.1016/j.sbi.2005.10.008 16263268

[B13] Doyle R. J. Chaloupka J. Vinter V. (1988). Turnover of cell walls in microorganisms. *Microbiol. Rev.* 52 554–567. 3070324 10.1128/mr.52.4.554-567.1988 PMC373163

[B14] Foster S. J. (1995). Molecular characterization and functional analysis of the major autolysin of *Staphylococcus aureus* 8325/4. *J. Bacteriol.* 177 5723–5725. 10.1128/jb.177.19.5723-5725.1995 7559367 PMC177389

[B15] Frankel M. B. Hendrickx A. P. Missiakas D. M. Schneewind O. (2011). LytN, a murein hydrolase in the cross-wall compartment of *Staphylococcus aureus*, is involved in proper bacterial growth and envelope assembly. *J. Biol. Chem.* 286 32593–32605. 10.1074/jbc.M111.258863 21784864 PMC3173183

[B16] Fuchs R. Stoehr P. Rice P. Omond R. Cameron G. (1990). New services of the EMBL data library. *Nucleic Acids Res.* 18 4319–4323. 10.1093/nar/18.15.4319 2388823 PMC331247

[B17] Gally D. Archibald A. R. (1993). Cell wall assembly in *Staphylococcus aureus*: proposed absence of secondary crosslinking reactions. *J. Gen. Microbiol.* 139 1907–1913. 10.1099/00221287-139-8-1907 8409927

[B18] Gisin J. Schneider A. Nagele B. Borisova M. Mayer C. (2013). A cell wall recycling shortcut that bypasses peptidoglycan de novo biosynthesis. *Nat. Chem. Biol.* 9 491–493. 10.1038/nchembio.1289 23831760

[B19] Götz F. Heilmann C. Stehle T. (2014). Functional and structural analysis of the major amidase (Atl) in Staphylococcus. *Int. J. Med. Microbiol.* 304 156–163. 10.1016/j.ijmm.2013.11.006 24444718

[B20] Hengstenberg W. Kohlbrecher D. Witt E. Kruse R. Christiansen I. Peters D. (1993). Structure and function of proteins of the phosphotransferase system and of 6-phospho-beta-glycosidases in Gram-positive bacteria. *FEMS Microbiol. Rev.* 12 149–163. 8398213 10.1111/j.1574-6976.1993.tb00016.x

[B21] Hill A. D. Reilly P. J. (2008). Computational analysis of glycoside hydrolase family 1 specificities. *Biopolymers* 89 1021–1031. 10.1002/bip.21052 18615662

[B22] Hiramatsu K. Katayama Y. Matsuo M. Sasaki T. Morimoto Y. Sekiguchi A. (2014). Multi-drug-resistant *Staphylococcus aureus* and future chemotherapy. *J. Infect. Chemother.* 20 593–601. 10.1016/j.jiac.2014.08.001 25172776

[B23] Kajimura J. Fujiwara T. Yamada S. Suzawa Y. Nishida T. Oyamada Y. (2005). Identification and molecular characterization of an N-acetylmuramyl-L-alanine amidase Sle1 involved in cell separation of *Staphylococcus aureus*. *Mol. Microbiol.* 58 1087–1101. 10.1111/j.1365-2958.2005.04881.x 16262792

[B24] Katoh K. Standley D. M. (2013). MAFFT multiple sequence alignment software version 7: improvements in performance and usability. *Mol. Biol. Evol.* 30 772–780. 10.1093/molbev/mst010 23329690 PMC3603318

[B25] Komatsuzawa H. Fujiwara T. Nishi H. Yamada S. Ohara M. Mccallum N. (2004). The gate controlling cell wall synthesis in *Staphylococcus aureus*. *Mol. Microbiol.* 53 1221–1231. 10.1111/j.1365-2958.2004.04200.x 15306023

[B26] Letunic I. Bork P. (2016). Interactive tree of life (iTOL) v3: an online tool for the display and annotation of phylogenetic and other trees. *Nucleic Acids Res.* 44 W242–W245. 10.1093/nar/gkw290 27095192 PMC4987883

[B27] Litzinger S. Duckworth A. Nitzsche K. Risinger C. Wittmann V. Mayer C. (2010a). Muropeptide rescue in *Bacillus subtilis* involves sequential hydrolysis by beta-N-acetylglucosaminidase and N-acetylmuramyl-L-alanine amidase. *J. Bacteriol.* 192 3132–3143. 10.1128/JB.01256-09 20400549 PMC2901692

[B28] Litzinger S. Fischer S. Polzer P. Diederichs K. Welte W. Mayer C. (2010b). Structural and kinetic analysis of *Bacillus subtilis* N-acetylglucosaminidase reveals a unique Asp-His dyad mechanism. *J. Biol. Chem.* 285 35675–35684. 10.1074/jbc.M110.131037 20826810 PMC2975192

[B29] Litzinger S. Mayer C. (2010). “Chapter 1: the murein sacculus,” in *Prokaryotic Cell Wall Compounds - Structure and Biochemistry*, eds König H. Claus H. Varma A. (New York, NY: Springer), 3–52.

[B30] Meeske A. J. Riley E. P. Robins W. P. Uehara T. Mekalanos J. J. Kahne D. (2016). Seds proteins are a widespread family of bacterial cell wall polymerases. *Nature* 537 634–638. 10.1038/nature19331 27525505 PMC5161649

[B31] Mokhtari A. Blancato V. S. Repizo G. D. Henry C. Pikis A. Bourand A. (2013). *Enterococcus faecalis* utilizes maltose by connecting two incompatible metabolic routes via a novel maltose 6′-phosphate phosphatase (MapP). *Mol. Microbiol.* 88 234–253. 10.1111/mmi.12183 23490043 PMC3633101

[B32] Monteiro J. M. Fernandes P. B. Vaz F. Pereira A. R. Tavares A. C. Ferreira M. T. (2015). Cell shape dynamics during the staphylococcal cell cycle. *Nat. Commun.* 6:8055. 10.1038/ncomms9055 26278781 PMC4557339

[B33] Postma P. W. Lengeler J. W. Jacobson G. R. (1993). Phosphoenolpyruvate:carbohydrate phosphotransferase systems of bacteria. *Microbiol. Rev.* 57 543–594. 8246840 10.1128/mr.57.3.543-594.1993 PMC372926

[B34] Ramadurai L. Lockwood K. J. Nadakavukaren M. J. Jayaswal R. K. (1999). Characterization of a chromosomally encoded glycylglycine endopeptidase of *Staphylococcus aureus*. *Microbiology* 145(Pt 4), 801–808. 10.1099/13500872-145-4-801 10220159

[B35] Reith J. Mayer C. (2011). Peptidoglycan turnover and recycling in Gram-positive bacteria. *Appl. Microbiol. Biotechnol.* 92 1–11. 10.1007/s00253-011-3486-x 21796380

[B36] Schaub R. E. Dillard J. P. (2017). Digestion of peptidoglycan and analysis of soluble fragments. *Bio Protoc.* 7:e2438. 10.21769/BioProtoc.2438 28932761 PMC5602577

[B37] Staedtler P. Hoenig S. Frank R. Withers S. G. Hengstenberg W. (1995). Identification of the active-site nucleophile in 6-phospho-beta-galactosidase from *Staphylococcus aureus* by labelling with synthetic inhibitors. *Eur. J. Biochem.* 232 658–663. 10.1111/j.1432-1033.1995.tb20857.x 7556220

[B38] Stamatakis A. (2014). RAxML version 8: a tool for phylogenetic analysis and post-analysis of large phylogenies. *Bioinformatics* 30 1312–1313. 10.1093/bioinformatics/btu033 24451623 PMC3998144

[B39] Stapleton M. R. Horsburgh M. J. Hayhurst E. J. Wright L. Jonsson I. M. Tarkowski A. (2007). Characterization of IsaA and SceD, two putative lytic transglycosylases of *Staphylococcus aureus*. *J. Bacteriol.* 189 7316–7325. 10.1128/JB.00734-07 17675373 PMC2168438

[B40] Sugai M. Komatsuzawa H. Akiyama T. Hong Y. M. Oshida T. Miyake Y. (1995). Identification of endo-β-N-acetylglucosaminidase and N-acetylmuramyl-L-alanine amidase as cluster-dispersing enzymes in *Staphylococcus aureus*. *J. Bacteriol.* 177 1491–1496. 10.1128/jb.177.6.1491-1496.1995 7883705 PMC176764

[B41] Sychantha D. Jones C. S. Little D. J. Moynihan P. J. Robinson H. Galley N. F. (2017). In vitro characterization of the antivirulence target of Gram-positive pathogens, peptidoglycan O-acetyltransferase A (OatA). *PLoS Pathog.* 13:e1006667. 10.1371/journal.ppat.1006667 29077761 PMC5697884

[B42] Takahashi J. Komatsuzawa H. Yamada S. Nishida T. Labischinski H. Fujiwara T. (2002). Molecular characterization of an atl null mutant of *Staphylococcus aureus*. *Microbiol. Immunol.* 46 601–612. 10.1111/j.1348-0421.2002.tb02741.x 12437027

[B43] Vollmer W. Blanot D. De Pedro M. A. (2008). Peptidoglycan structure and architecture. *FEMS Microbiol. Rev.* 32 149–167. 10.1111/j.1574-6976.2007.00094.x 18194336

[B44] Wheeler R. Turner R. D. Bailey R. G. Salamaga B. Mesnage S. Mohamad S. A. (2015). Bacterial cell enlargement requires control of cell wall stiffness mediated by peptidoglycan hydrolases. *mBio* 6:e00660. 10.1128/mBio.00660-15 26220963 PMC4551982

[B45] Witt E. Frank R. Hengstenberg W. (1993). 6-Phospho-beta-galactosidases of gram-positive and 6-phospho-beta-glucosidase B of gram-negative bacteria: comparison of structure and function by kinetic and immunological methods and mutagenesis of the *lacG* gene of *Staphylococcus aureus*. *Protein Eng.* 6 913–920. 10.1093/protein/6.8.913 8309940

[B46] Wong W. Young F. E. Chatterjee A. N. (1974). Regulation of bacterial cell walls: turnover of cell wall in *Staphylococcus aureus*. *J. Bacteriol.* 120 837–843. 4455686 10.1128/jb.120.2.837-843.1974 PMC245846

[B47] Yamada S. Sugai M. Komatsuzawa H. Nakashima S. Oshida T. Matsumoto A. (1996). An autolysin ring associated with cell separation of *Staphylococcus aureus*. *J. Bacteriol.* 178 1565–1571. 10.1128/jb.178.6.1565-1571.1996 8626282 PMC177839

[B48] Yip V. L. Thompson J. Withers S. G. (2007). Mechanism of GlvA from *Bacillus subtilis*: a detailed kinetic analysis of a 6-phospho-alpha-glucosidase from glycoside hydrolase family 4. *Biochemistry* 46 9840–9852. 10.1021/bi700536p 17676871

