# Supplementary material for: Recovery of the Peptidoglycan Turnover Product Released by the Autolysin Atl in Staphylococcus aureus Involves the Phosphotransferase System Transporter MurP and the Novel 6-phospho-N-acetylmuramidase MupG
Source: Front Microbiol. 2018 Nov 16;9:2725. doi: 10.3389/fmicb.2018.02725 (PMC6262408; doi:10.3389/fmicb.2018.02725)
Supplement: Supplementary file 1 [file Data_Sheet_1.pdf]

Supplementary Material

Recovery of the Peptidoglycan Turnover Product Released by the Autolysin Atl in *Staphylococcus aureus* Involves the Phosphotransferase System Transporter MurP and the Novel 6-phospho-*N*-acetylmuramidase MupG

Robert Maria Kluj, Patrick Ebner, Martina Adamek, Nadine Ziemert, Christoph Mayer* and Marina Borisova*

*** Correspondence:** Christoph Mayer: christoph.mayer@uni-tuebingen.de
Marina Borisova: marina.borisova@uni-tuebingen.de

1 Supplementary Data

Generation of markerless Δ*mupG* and Δ*mupGmurQ* mutants

The markerless *S. aureus* USA300 JE2 Δ*mupG* and Δ*mupG*Δ*murQ* mutants were generated by amplifying an about 1000 bp fragment upstream of *SAUSA300_0192* (*mupG*) gene (primer pair RK-2 and RK-3 for Δ*mupG* and primer pair RK-2 and RK-6 for *Sa* Δ*mupGmurQ*; see supplementary Table 1 for a complete list of primers) and an about 1100 bp fragment downstream of *mupG* (primers RK-4 and RK-5 for Δ*mupG* and RK-7 and RK-8 for Δ*mupGmurQ*) using genomic DNA. Shuttle vector pBASE6 (Geiger et al., 2012) was linearized with EcoRV and the three fragments were ligated by Gibson assembly (Gibson et al., 2009). Chemically competent *E. coli* DH5α cells, growing on LB with ampicillin were controlled for correct pBASE6-*mupG*_del and pBASE6-*mupGmurQ*_del plasmid DNA sequence by colony PCR and sequencing (MWG Eurofins). Furthermore, *E. coli*

DC10B cells that lack *dcm* methylation and therefore allow direct plasmid transfer into *S. aureus*, were transformed with pBASE6-*mupG*_del and pBASE6-*mupGmurQ*_del plasmids and successful transformants were selected by growth on LB with ampicillin.

Electrocompetent *S. aureus* cells were transformed as described (Löfblom et al., 2007) with 1 - 2 µg DNA (1 mm cuvette, 23 kV/cm) and after electroporation, warm BM medium was added to the cells. Transformed cells were further incubated for 2 h at 37°C and finally plated on BM agar supplemented with 10 µg/ml chloramphenicol.

The suicide-integration vector pBASE6 allows anhydrotetracycline-inducible expression of *secY* antisense RNA for counter-selection against the plasmid (Geiger et al., 2012). The deletion of *mupG* respectively *mupGmurQ* was further conducted as described previously (Bae and Schneewind, 2006;Geiger et al., 2012). The correct sequence of the obtained markerless mutants was verified by colony PCR and sequencing.

MupG complementation

S. aureus USA300 JE2 Δ*mupG* mutant was complemented *in trans* using the *E. coli* - *S. aureus* shuttle vector pCtufamp (Ebner et al., 2015), which constitutively expresses the *mupG* gene under control of the *tufA* promoter. The gene *mupG* was amplified from USA300 JE2 genomic DNA using primers RK-26 and RK-27. The pCtufamp plasmid was linearized with PacI and HindIII restriction enzyme and cloning with the *mupG* PCR product was acchieved using Gibson assembly (Gibson et al., 2009). Successful *E. coli* DH5α pCtufamp-*mupG* transformants were selected by ampicillin resistance and the correct DNA sequence of the plasmid was confirmed by restriction analysis and sequencing. Plasmid pCtufamp-*mupG* was isolated from DH5α cells and further introduced in DC10B cells to allow direct transformation of DNA from *E. coli* into *S. aureus* USA300 Δ*mupG* mutant strain. Electroporation of the plasmid pCtufamp-*mupG* into *S. aureus* cells was performed as previously described in the material and method section. *S. aureus* transformants were selected by chloramphenicol resistance and the presence of the complementation plasmid was verified by colony PCR.

2 Supplementary Figures and Tables

2.1 Supplementary Figures

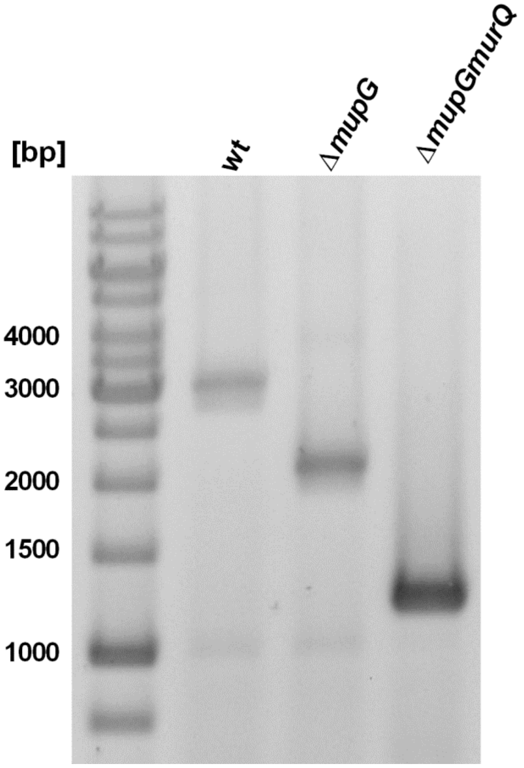

Supplementary Figure 1. PCR controls of mutants, generated in this study. Genomic DNA was isolated from *S. aureus* JE2 wild type strain (wt), Δ*mupG* (formerly designated as *S. aureus* Δ*SAUSA_0192*) cells, as well from Δ*mupGmurQ* (Δ*SAUSA_0192–0193)* cells. Chromosomal regions of interest were amplified by PCR, using primer pair RK-2/RK-5 listed in the Supplementary Table 1 of the supplementary material, and the expected sizes of the PCR products in base pairs (bp) were as indicated: wt - 3137 bp, Δ*mupG* - 2105 bp, and Δ*mupGmurQ* - 1200 bp.

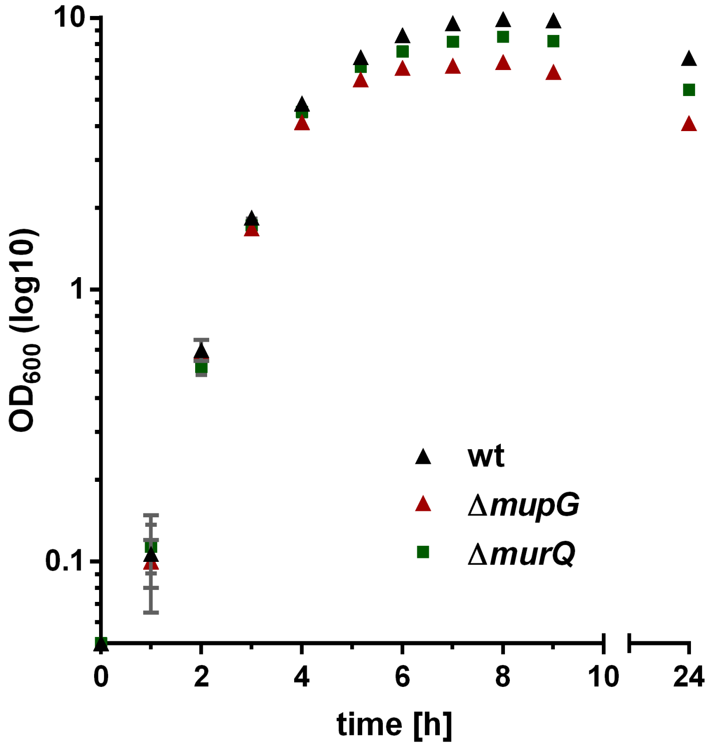

Supplementary Figure 2. Growth kinetics of *S. aureus* wild type and peptidoglycan recycling mutants in LB medium. Wild type (wt, black triangles), Δ*mupG* mutant (red triangle), and Δ*murQ* mutant cells (green squares) were grown in LB medium under constant shaking and the optical density was monitored at 600 nm (OD_{600}) for up to 24 h. The experiment was performed in triplicates and data were presented in Prism 6 as mean ± SD.

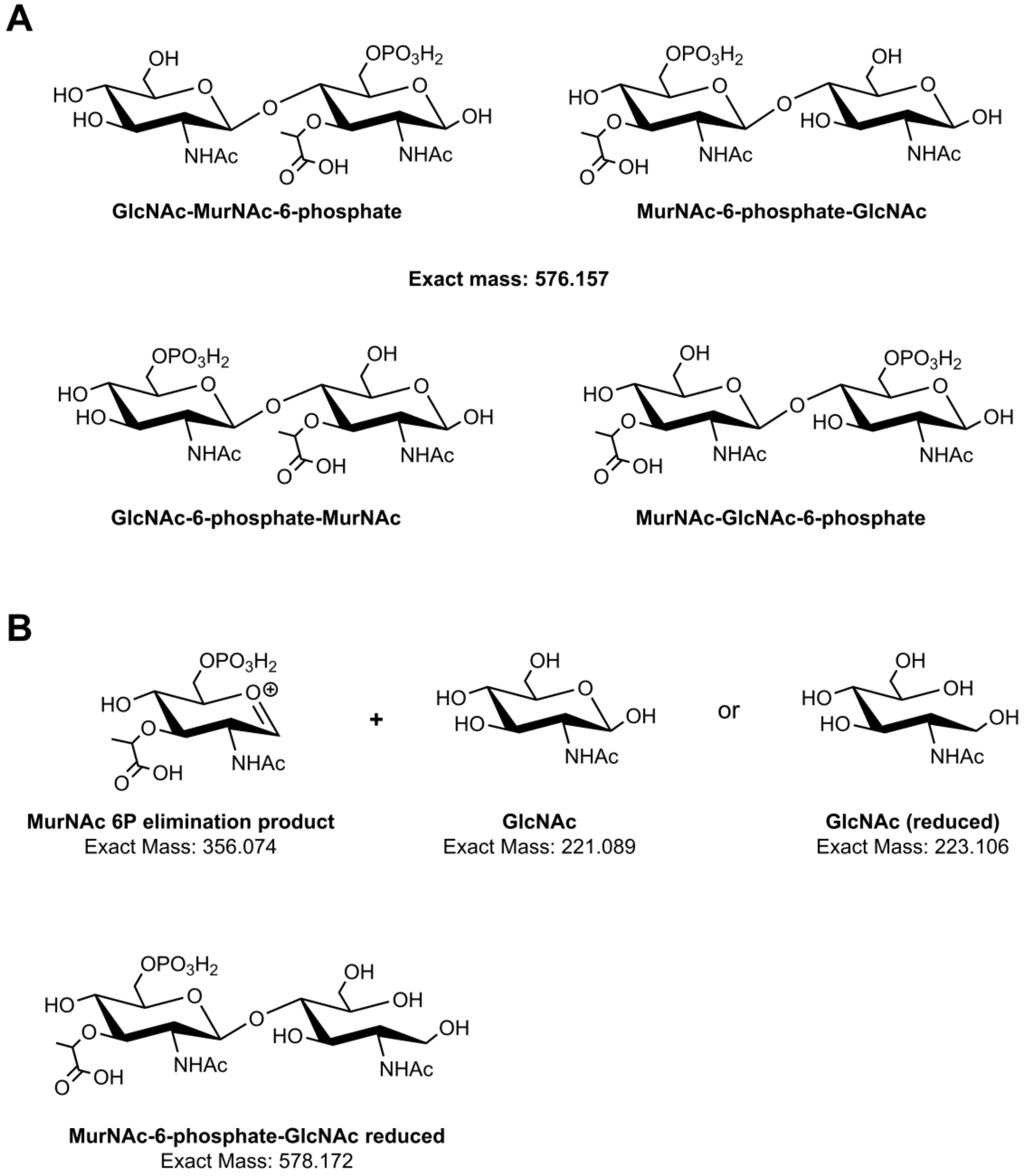

Supplementary Figure 3. Chemical structures of phosphorylated disaccharides composed of GlcNAc and MurNAc and MS fragmentation products. (A) A compound accumulates in Δ*mupG* cells with a monoisotopic molecular mass (exact mass) of 576.157 Da, which corresponds to a phosphorylated disaccharide consisting of GlcNAc and MurNAc sugars. Reduction of the compound with $NaBH_4$ at the reducing end indicated that the disaccharide is not phosphorylated at position C1. Assuming phosphorylation at the C6 position, the depicted four different structures are possible structures of the compound. (B) Obtained mass spectra fragmentation pattern of MurNAc 6P-GlcNAc, after reduction of the Δ*mupG* cytosolic fraction with $NaBH_4$ and structure of MurNAc 6P-GlcNAc after reduction with $NaBH_4$.

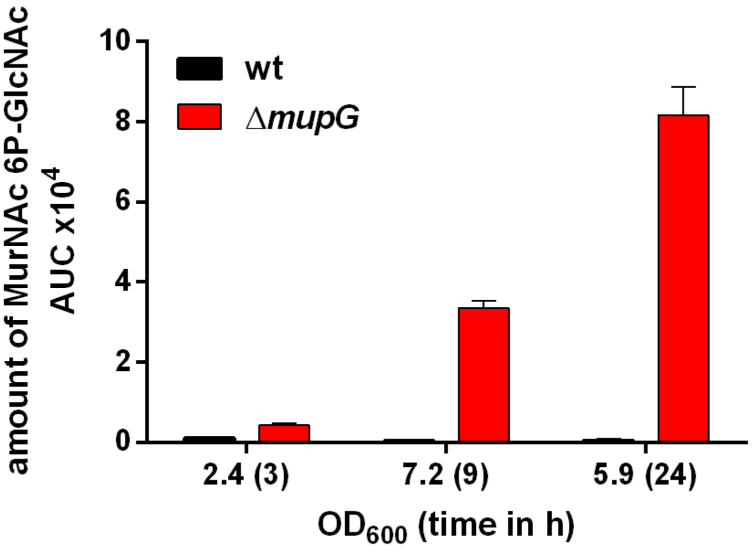

Supplementary Figure 4. Growth phase-dependent accumulation of MurNAc 6P-GlcNAc. *S. aureus* wild type (wt) and Δ*mupG* cells were grown in LB medium for 3 h (OD_{600} of ~ 2.4), 9 h (OD_{600} of ~ 7.2) and 24 h (OD_{600} of ~ 5.9) and the same cell number was adjusted for all growth phases. Cytosolic fractions were generated and analyzed by LC-MS in negative ion-mode. Based on the extracted ion chromatographs (EIC) for MurNAc 6P-GlcNAc (observed mass of $(M+H)^-$ = 575.149 m/z), amounts of this compound were determined by calculating the area under the curve (AUC) for the corresponding EIC.

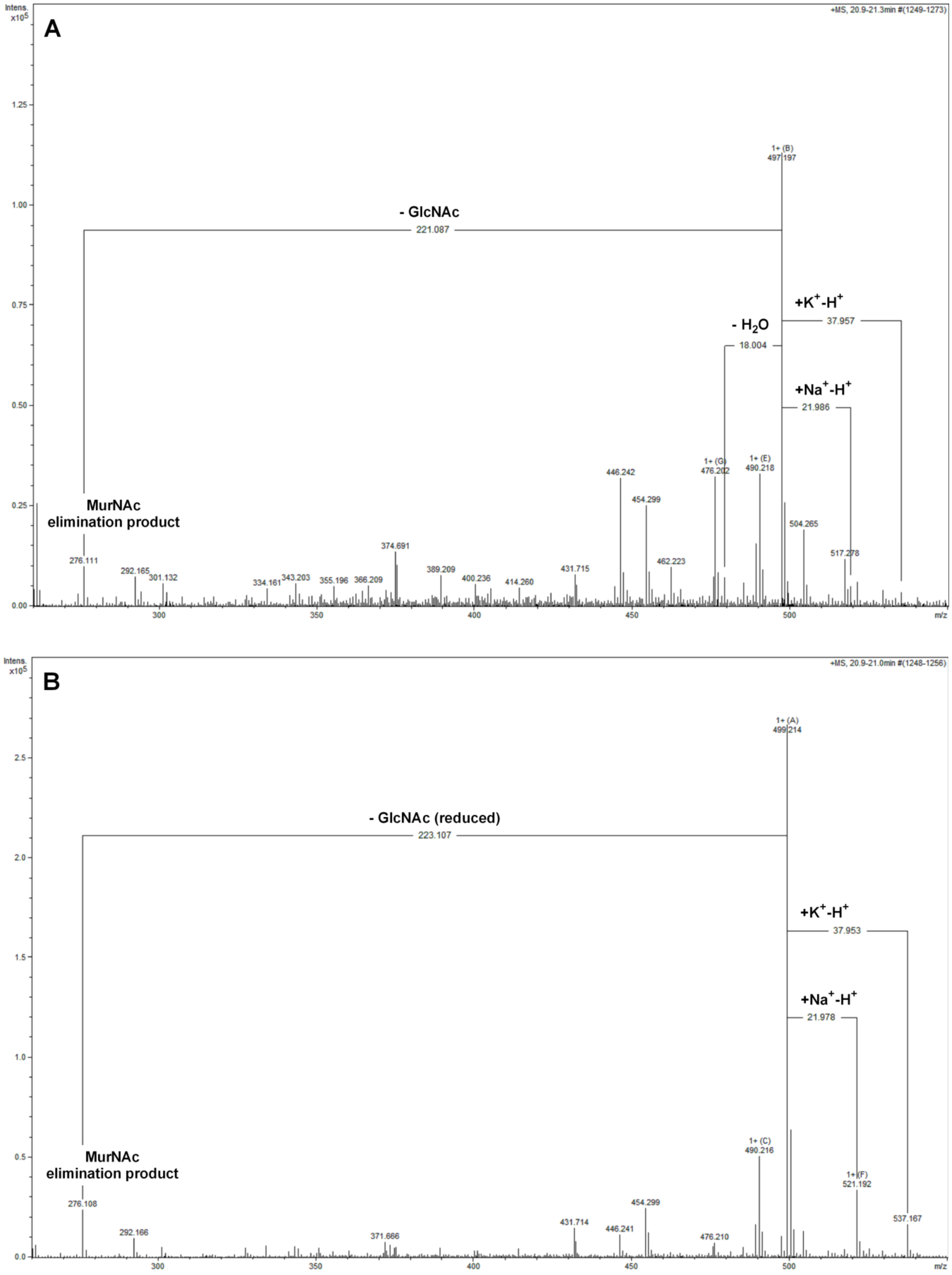
A
Intens. x10^5
+MS, 20.9-21.3min #(1249-1273)
1+ (B) 497.197
- GlcNAc
221.087
+K+-H+
37.957
- H2O
18.004
+Na+-H+
21.986
MurNAc elimination product
276.111
292.165
301.132
334.161
343.203
355.196
366.209
374.691
389.209
400.236
414.260
431.715
446.242
454.299
462.223
1+ (G) 476.202
1+ (E) 490.218
504.265
517.278
m/z
B
Intens. x10^5
+MS, 20.9-21.0min #(1248-1256)
1+ (A) 499.214
- GlcNAc (reduced)
223.107
+K+-H+
37.953
+Na+-H+
21.978
MurNAc elimination product
276.108
292.166
371.666
431.714
446.241
454.299
476.210
1+ (C) 490.216
1+ (F) 521.192
537.167
m/z

Supplementary Figure 5. Accumulation of MurNAc-GlcNAc in the growth medium of MurP transporter mutants. *S. aureus* wild type (wt), *murP* transporter mutant (*murP::Tn*) and *murQ*-operon mutant (Δ*mupGmurQPR*) were cultured to stationary phase and the culture medium was prepared prior to LC-MS measurement (positive ion-mode) as described in the legend of figure 6**.** The culture medium was analyzed for the accumulation of compounds present in both mutants, which were absent in the wt strain. A compound with an observed mass of $(M+H)^+$ = 497.197 m/z accumulates specifically in the supernatant of *mupP::Tn* cells and with an observed mass of 497.195 m/z in the *murQ*-operon cells. After reduction of both samples with $NaBH_4$, this compound diminished and a new compound with a mass of $(M+H)^+$ = 499.214 m/z was generated. The mass spectra fragmentation pattern and adducts for the compound before and after reduction, presented in Compass DataAnalysis program (Bruker) from 200 to 600 m/z, indicated that the culture supernantant of both MurP transporter mutants contains MurNAc-GlcNAc and not GlcNAc-MurNAc disaccharide.

2.2 Supplementary Tables

Supplementary Table 1. Oligonucleotides used in this study

Primer	Sequence (5'-3')	Application
RK-2	cgcgcagatctgtcgacgatatCACCTGTTTGTAATGTTGGG	deletion of *mupG* and *mupGmurQ*
RK-3	actccctagtCAAAGTAGCCCTCCTTAACAATATAATTATTAG	deletion of *mupG*
RK-4	ggctactttgACTAGGGAGTGTAAGAAGTG	deletion of *mupG*
RK-5	tgcaggcatgcaagcttgatatCGTGTATAACACCCATGACAC	deletion of *mupG*
RK-6	aaggttgtctCAAAGTAGCCCTCCTTAACAATATAATTATTAG	deletion of *mupGmurQ*
RK-7	ggctactttgAGACAACCTTAGGAGGGATTTAAATGACCAAAG	deletion of *mupGmurQ*
RK-8	tgcaggcatgcaagcttgataTCCAGCACCAGCCATCGCTG	deletion of *mupGmurQ*
RK-26	agagttcgaggaggtttaattaaTGACAGGCTTTTCAGTGTATTTAG	cloning of *mupG* in pCtufamp
RK-27	gtctattattaagtacttcagctaattaagcttTCACTTCTTACACTCCCTAG	cloning of *mupG* in pCtufamp
MB-67	ggccatgggaACAGGCTTTTCAGTGTATTTAGGA	cloning of *mupG* in pET28a
MB-68	ggctcgagCTTCTTACACTCCCTAGTTT	cloning of *mupG* in pET28a

Recognitions sites of restriction enzymes underlined

Supplementary Table 2. Plasmids and strains used in the study

Strains and plasmids	Description/Application	Reference/Source
Plasmids		
pBASE6	*Escherichia coli* - *Staphylococccus aureus* temperature-sensitive suicide shuttle vector, Amp^r, Cam^r	(Geiger et al., 2012)
pBASE6-*mupG*_del	pBASE suicide integration vector for deletion of *SAUSA300_0192* (*mupG*), Amp^r, Cam^r	this study
pBASE6-*mupGmurQ*_del	pBASE6 suicide integration vector for deletion of *SAUSA300_0192* (*mupG*) and *SAUSA300_0193* (*murQ*), Amp^r, Cam^r	this study
pET28a (+)	*E. coli* expression vector, C-terminal 6x His, Kan^r	Novagen
pET28a-*mupG*	Cloning of *SAUSA300_0192* (*mupG*) in pET28a, Kan^r	this study
pCtufamp	*E. coli* - *S. aureus* shuttle vector, Amp^r, Cam^r	(Ebner et al., 2015)
pCtufamp-*mupG*	Cloning of *SAUSA300_0192* (*mupG*) in pCtufamp, Amp^r, Cam^r	this study
Strains		
E. coli DH5α	*SupE44 hsdR17 recA1 endA1 gyrA96 thi-1 relA1*	(Hanahan, 1983)
E. coli DH5α pET28a-*mupG*	DH5α cells transformed with pET28a-*mupG,* Kan^r	this study
E. coli DH5α pCtufamp-*mupG*	DH5α cells transformed with pCtufamp-*mupG*, Amp^r	this study
E. coli BL21(DE3)	F$^-$ *ompT hsdS*$_B$($r_B^-m_B^-$) *gal dcm* (DE3)	(Studier and Moffat, 1986)
E. coli BL21(DE3) pET28a-*mupG*	Heterologous expression of recombinant *S. aureus* USA300 JE2 *mupG*, Kan^r	this study
E. coli DC10B	*E. coli* DH10B Δ*dcm*	(Monk et al., 2012)
E. coli DC10B pBASE6	DC10B cells transformed with pBASE6, Amp^r	Friedrich Götz laboratory
E. coli DC10B pBASE6-*mupG*_del	DC10B cells transformed with pBASE6-*mupG*_del, Amp^r	this study
E. coli DC10B pBASE6-*mupGmurQ*_del	DC10B cells transformed with pBASE6-*mupGmurQ*_del, Amp^r	this study
E. coli DC10B pCtufamp	DC10B cells transformed with pCtufamp, Amp^r	(Ebner et al., 2015)
E. coli DC10B pCtufamp-*mupG*	DC10B cells transformed with pCtufamp-*mupG*, Amp^r	this study
S. aureus USA300 JE2	USA300 LAC, cured for 3 plasmids for antibiotic resistance, parental strain	(Fey et al., 2013)
S. aureus Δ*mupG*	Markerless *SAUSA300_0192* (*mupG*) deletion mutant of JE2	this study
S. aureus Δ*mupGmurQ*	Markerless *SAUSA300_0192* (*mupG*) and *SAUSA300_0193* (*murQ*) deletion mutant of JE2	this study
S. aureus murP::Tn	*Bursa aurealis* transposon *SAUSA300_0194* (*murP*) insertion mutant of JE2	(Fey et al., 2013)
S. aureus Δ*murQ*	Markerless *SAUSA300_0193* deletion mutant of JE2	(Borisova et al., 2016)
S. aureus Δ*mupGmurQPR* (Δ*murQ*-op)	Markerless *SAUSA300_0192-SAUSA300_0195* deletion mutant of JE2	(Borisova et al., 2016)
S. aureus Δ*mupG* pCtufamp	JE2 Δ*mupG* transformed with pCtufamp, Cam^r	this study
S. aureus Δ*mupG* pCtufamp-*mupG*	JE2 Δ*mupG* transformed with pCtufamp-*mupG,* Cam^r	this study

Supplementary Table 3. List of species containing putative MupG orthologous and MupG-like proteins (included in the phylogenetic tree, Figure 8 main text)

Tax ID	Scientific name of species	Accession number (uniprot ID)	Protein length (AA)
592010	*Abiotrophia defectiva* ATCC 49176	W1Q1H1	342
1009370	*Acetonema longum* DSM 6540	F7NMV1	363
		F7NNS1	361
591001	*Acidaminococcus fermentans* DSM 20731	D2RNC1	356
568816	*Acidaminococcus intestini* RyC-MR95	G4Q715	354
933801	*Acidianus hospitalis* W1	F4B5C6	211
		F4B5E0	345
111015	*Actinomyces radicidentis*	A0A0X8JDX3	350
866775	*Aerococcus urinae* ACS-120-V-Col10a	F2I6S4	362
655812	*Aerococcus viridans* ATCC 11563 = CCUG 4311	D4YE07	353
1229520	*Alkalibacterium* sp. AK22	A0A011QTR6	361
525254	*Anaerococcus lactolyticus* ATCC 51172	C2BGB9	361
525919	*Anaerococcus prevotii* DSM 20548	C7REF0	329
655811	*Anaerococcus vaginalis* ATCC 51170	C7HW89	332
861450	*Anaeroglobus geminatus* F0357	G9YK66	359
1794912	*Anaerosporomusa subterranea*	A0A154BSE5	358
82374	*Anaerovibrio lipolyticus*	A0A0B2JRX6	359
1490057	*Anoxybacillus* sp. B7M1	A0A160FCK4	357
997872	*Atopobium minutum* 10063974	N2BKM7	364
521095	*Atopobium parvulum* DSM 20469	C8W835	351
		C8W890	364
553184	*Atopobium rimae* ATCC 49626	B9CLC8	368
525256	*Atopobium vaginae* DSM 15829	F1T5I2	365
		F1T6R7	360
866774	*Atopobium vaginae* PB189-T1-4	E1L2A4	381
1637974	*Bacilli bacterium* VT-13-104	A0A0K0G9A4	360
1392	*Bacillus anthracis*	Q81UN9	354
		Q81UR7	361
		Q81US3	362
1117379	*Bacillus bataviensis* LMG 21833	K6E9W9	335
1408103	*Bacillus campisalis*	A0A0M2T4I8	346
226900	*Bacillus cereus* ATCC 14579	Q81HG9	357
		Q81HJ5	361
		Q81HK1	362
526986	*Bacillus cereus* Rock3-44	C2W4D9	362
		C2WFY5	363
526988	*Bacillus cereus* Rock4-18	C2WYP2	362
		C2WYP7	361

		C2WYS6	353
1397	*Bacillus circulans*	A0A0J1IPX4	337
66692	*Bacillus clausii* KSM-K16	Q5WBR3	361
		Q5WLA0	352
345219	*Bacillus coagulans* 36D1	G2THA2	361
		G2TKE3	359
315749	*Bacillus cytotoxicus* NVH 391-98	A7GLJ0	362
		A7GLL6	356
1402860	*Bacillus enclensis*	A0A0V8HQC0	356
272558	*Bacillus halodurans* C-125	Q9K701	351
246786	*Bacillus indicus*	A0A084H2K9	336
1367477	*Bacillus infantis* NRRL B-14911	U5LE36	337
284581	*Bacillus koreensis*	A0A0M0KWI1	336
1246626	*Bacillus lehensis* G1	A0A060LN41	350
1679170	*Bacillus loiseleuriae*	A0A0K9GRV8	358
		A0A0K9GW51	361
574376	*Bacillus manliponensis*	A0A073JVS4	355
189381	*Bacillus marisflavi*	A0A0M0G1B4	337
		A0A0M0G3W2	363
545693	*Bacillus megaterium* QM B1551	D5DXW9	362
		D5E232	336
398511	*Bacillus pseudofirmus* OF4	D3FRN2	360
1321606	*Bacillus selenatarsenatis* SF-1	A0A0A8X7B9	361
157838	*Bacillus shackletonii*	A0A0Q3WSN9	355
1479	*Bacillus smithii*	A0A0H4P0T1	357
665959	*Bacillus* sp. 2_A_57_CT2	E5WFS1	355
1476857	*Bacillus* sp. B-jedd	A0A098F632	354
1581033	*Bacillus* sp. FJAT-21945	A0A0M0WAQ0	361
1581037	*Bacillus* sp. FJAT-22058	A0A0M1NM28	336
		A0A0M1NN47	360
		A0A0M1NXK1	367
1712029	*Bacillus* sp. FJAT-25509	A0A0Q3SIR4	364
		A0A0Q3SJA6	362
		A0A0Q3TR97	375
		A0A0Q3VY92	363
		A0A0Q3WA13	354
1460639	*Bacillus* sp. JCM 19045	W7Z9H7	352
1811976	[*Bacillus*] sp. KCTC 13219	A0A150YL91	360
1455638	*Bacillus* sp. SA1-12	A0A0M2PAQ4	339
1414648	*Bacillus* sp. SGD-V-76	A0A0V8JJX2	337
1423321	*Bacillus* sp. SJS	A0A176J169	338
46224	*Bacillus sporothermodurans*	A0A150KK53	362
		A0A150L6B6	355
35841	*Bacillus thermoamylovorans*	A0A090IYV8	357
218284	*Bacillus vietnamensis*	A0A0P6VV75	365
		A0A0P6WPF6	343

1131730	*Bacillus vireti* LMG 21834	W1SAN2	333
		W1SH02	362
1235279	*Bhargavaea cecembensis* DSE10	M7NA07	359
1432657	*Borrelia duttonii* CR2A	W6TX00	365
224326	*Borreliella burgdorferi* B31	O50983	365
1262760	*Brachyspira* sp. CAG:700	R5LFE6	360
		R5M021	360
1042163	*Brevibacillus laterosporus* LMG 15441	A0A075RA01	362
1265861	*Brochothrix campestris* FSL F6-1037	W7CPL6	348
		W7CQR3	361
1121126	*Brochothrix thermosphacta* DSM 20171 = FSL F6-1036	W7CBN2	348
		W7CL20	362
301148	*Caldibacillus debilis*	A0A150MCP7	354
397948	*Caldivirga maquilingensis* IC-167	A8MAC8	356
1714254	*Caldivirga* sp. JCHS_4	A0A101XFL3	356
1156417	*Caloranaerobacter azorensis* H53214	A0A096CWF3	363
1029718	*Candidatus Arthromitus* sp. SFB-mouse-Japan	F9VK14	364
1574263	*Candidatus Stoquefichus* sp. KLE1796	A0A139T806	362
		A0A139TFC3	383
		A0A139TL24	362
1449336	*Carnobacterium divergens* DSM 20623	A0A0R2HWL4	362
		A0A0R2HXR1	363
		A0A0R2HYC1	355
1266845	*Carnobacterium inhibens* subsp. *gilichinskyi*	U5S9F2	358
1234679	*Carnobacterium maltaromaticum* LMA28	K8E2H1	354
		K8E3C8	366
		K8EEA3	362
		K8EHX2	363
		K8EUW3	362
1564681	*Carnobacterium* sp. CP1	A0A0U3E833	361
		A0A0U3PMV6	354
1234409	*Catellicoccus marimammalium* M35/04/3	K8Z886	363
1262767	*Catenibacterium* sp. CAG:290	R7GHT6	363
1319815	*Cetobacterium somerae* ATCC BAA-474	U7V6E0	361
1734399	*Clostridia bacterium* BRH_c25	A0A101WH66	361
		A0A101WNU7	346
		A0A124IQ14	374
457421	*Clostridiales bacterium* 1_7_47FAA	C5EV19	364
1497955	*Clostridiales bacterium* KA00274	A0A133YCB3	366
1321778	*Clostridiales bacterium* oral taxon 876 str. F0540	U2DBE3	360
		U2DDN0	362
		U2ERE0	364
272563	*Clostridioides difficile* 630	Q181P6	364
		Q184N8	365
1418104	*Clostridium argentinense* CDC 2741	A0A0C1R1D8	360
518636	[*Clostridium*] *asparagiforme* DSM 15981	C0D5D6	379

		C0D9V7	365
1415775	*Clostridium baratii* str. Sullivan	A0A0A7FVD6	363
		A0A0A7FVZ5	359
290402	*Clostridium beijerinckii* NCIMB 8052	A6LUQ9	357
		A6LWT3	383
1263064	*Clostridium bolteae* CAG:59	R5F7I6	364
441771	*Clostridium botulinum* A str. Hall	A5I1I0	358
		A5I779	361
935198	*Clostridium botulinum* B str. Eklund 17B (NRP)	B2TJW3	364
		B2TPX0	348
632245	*Clostridium butyricum* E4 str. BoNT E BL5262	C4IBH5	360
		C4IEN6	362
545697	*Clostridium celatum* DSM 1785	L1QKV7	364
717608	[*Clostridium*] cf. *saccharolyticum* K10	D6DDJ6	363
1263065	*Clostridium clostridioforme* CAG:132	R6JX67	364
999411	*Clostridium colicanis* 209318	N9XJC8	365
		N9XYQ8	363
1263067	*Clostridium hathewayi* CAG:224	R5T0A6	356
1294142	*Clostridium intestinale* URNW	U2NJS4	362
610130	[*Clostridium*] *saccharolyticum* WM1	D9QZR8	349
		D9R406	364
		D9R9Q7	363
457396	*Clostridium* sp. 7_2_43FAA	V9H2C9	359
		V9HEP5	364
		V9HEV0	363
1354301	*Clostridium* sp. BL8	T0NCG8	362
641107	*Clostridium* sp. DL-VIII	G7MCB4	362
747377	*Clostridium* sp. DMHC 10	A0A0L8EYU2	364
1609975	*Clostridium* sp. FS41	A0A0F0CI11	371
1226325	*Clostridium* sp. KLE 1755	U2CQ75	361
1196322	*Clostridium* sp. Maddingley MBC34-26	K6TV16	364
1230342	*Clostridium tetanomorphum* DSM 665	A0A037Z936	361
1288971	[*Clostridium*] *ultunense* Esp	M1Z6D9	318
		M1ZI56	362
521003	*Collinsella intestinalis* DSM 13280	C4F7L5	361
		C4FB87	351
1262850	*Collinsella* sp. CAG:166	R5ZF55	350
445975	*Collinsella stercoris* DSM 13279	B6G9L1	361
		B6G9Q2	368
742742	*Collinsella tanakaei* YIT 12063	G1WIN1	351
100884	*Coprobacillus cateniformis*	E7G6Q3	358
		E7G898	363
		E7G9Z6	236
		E7GB41	383
		E7GE27	389
		E7GG55	364

1262853	*Coprobacillus* sp. CAG:183	R5R7X9	382
1262854	*Coprobacillus* sp. CAG:235	R5Q8K4	382
		R5QMK5	237
1262856	*Coprobacillus* sp. CAG:698	R5FU40	303
700015	*Coriobacterium glomerans* PW2	F2N9Q3	360
1736311	*Curtobacterium* sp. Leaf261	A0A0Q4V419	340
997346	*Desmospora* sp. 8437	F5SCB8	359
883103	*Dolosigranulum pigrum* ATCC 51524	H3NEY8	343
999415	*Eggerthia catenaformis* OT 569 = DSM 20559	M2NHB4	365
1158606	*Enterococcus asini* ATCC 700915	R2PPF2	363
		R2PYG1	363
		R2Q4V5	356
1140002	*Enterococcus avium* ATCC 14025	S0RQJ4	363
		S0RWQ6	352
		S0RWS1	360
565655	*Enterococcus casseliflavus* EC20	C9A4Q4	363
		C9A8H2	363
		C9A755	356
		C9A9T1	354
1121864	*Enterococcus cecorum* DSM 20682 = ATCC 43198	S1RPS0	362
1121865	*Enterococcus columbae* DSM 7374 = ATCC 51263	S0KY94	352
1139219	*Enterococcus dispar* ATCC 51266	S1N7Z2	363
		S1NYV2	357
1260356	*Enterococcus faecalis* 13-SD-W-01	S4CYV8	378
		S4D9R3	362
		S4DG08	353
226185	*Enterococcus faecalis* V583	Q831R3	369
		Q834U7	363
		Q837K1	362
333849	*Enterococcus faecium* DO	I3TZU2	353
		I3U1N4	364
1158608	*Enterococcus haemoperoxidus* ATCC BAA-382	R2QC85	362
		R2QQ37	364
		R2QWQ2	354
		R2TGU6	363
768486	*Enterococcus hirae* ATCC 9790	I6SER8	246
		I6SV66	358
		I6T3X3	362
		I6TD20	364
888064	*Enterococcus italicus* DSM 15952	E6LD00	354
1300150	*Enterococcus mundtii* QU 25	V5XMS1	363
		V5XQ78	355
		V5XSW8	362
1158607	*Enterococcus pallens* ATCC BAA-351	R2PXP5	362
		R2QLR1	380
1158610	*Enterococcus phoeniculicola* ATCC BAA-412	R3TMD4	362

		R3W9R5	362
		R3WWH7	364
1139996	*Enterococcus saccharolyticus* subsp. *saccharolyticus* ATCC 43076	S0NIG9	363
1316414	*Enterococcus* sp. HSIEG1	T0U472	308
		T0U7Y1	357
		T0VMC0	311
1140003	*Enterococcus sulfureus* ATCC 49903	S0LAJ5	362
		S0P1M4	361
908337	*Eremococcus coleocola* ACS-139-V-Col8	E4KR33	363
1514105	*Erysipelothrix larvae*	A0A0X8GZ09	362
		A0A0X8H0D6	360
		A0A109UHF6	361
		A0A109UHQ6	362
650150	*Erysipelothrix rhusiopathiae* str. Fujisawa	F5WSZ0	362
		F5WT23	345
		F5WTW6	362
658659	*Erysipelotrichaceae bacterium* 3_1_53	E2SNA2	361
1262981	*Erysipelotrichaceae bacterium* CAG:64	R6UPR9	361
		R6VXC8	364
1263076	*Eubacterium dolichum* CAG:375	R7G5E0	362
457402	*Eubacterium* sp. 3_1_31	H1BI77	361
		H1BM35	365
262543	*Exiguobacterium sibiricum* 255-15	B1YFD1	357
360911	*Exiguobacterium* sp. AT1b	C4KZ05	349
883112	*Facklamia ignava* CCUG 37419	K1ME59	350
718252	*Faecalibacterium prausnitzii* L2-6	D4K1I5	349
1702221	*Faecalibaculum rodentium*	A0A140DXV5	362
		A0A140DXW3	409
1017270	*Fictibacillus enclensis*	A0A0V8J9B2	347
1196324	*Fictibacillus macauensis* ZFHKF-1	I8UHR2	363
1221500	*Fictibacillus phosphorivorans*	A0A160IPL6	352
1263004	*Firmicutes bacterium* CAG:137	R6H0B3	355
1263017	*Firmicutes bacterium* CAG:313	R6YTX6	345
1235796	*Firmicutes bacterium* M10-2	R9LSC9	395
		R9LTA6	361
471223	*Geobacillus* sp. WCH70	C5DAG8	356
638301	*Granulicatella adiacens* ATCC 49175	C8NHI2	351
		C8NHJ0	363
626369	*Granulicatella elegans* ATCC 700633	D0BLW8	345
572479	*Halanaerobium praevalens* DSM 2228	E3DMW2	345
1033810	*Haloplasma contractile* SSD-17B	F7PUD1	351
883114	*Helcococcus kunzii* ATCC 51366	H3NM85	353
545696	*Holdemania filiformis* DSM 12042	B9Y2X9	382
220754	*Jeotgalibacillus campisalis*	A0A0C2VVB6	369
1410657	*Kandleria vitulina* DSM 20405	A0A0R2H9W0	365

1750719	*Kurthia* sp. 11kri321	A0A0X1RVR5	342
936594	*Lachnoanaerobaculum* sp. ICM7	J5GBB5	365
397290	*Lachnospiraceae bacterium* A2	R9K663	393
		R9K720	300
1600	*Lactobacillus acetotolerans*	A0A0D6A1V9	346
		A0A0D6A4Q2	364
272621	*Lactobacillus acidophilus* NCFM	Q5FHY1	352
		Q5FIF9	353
		Q5FJQ2	363
1423719	*Lactobacillus algidus* DSM 15638	A0A0R1HIN0	363
585524	*Lactobacillus amylolyticus* DSM 11664	D4YTG1	351
1423721	*Lactobacillus amylophilus* DSM 20533 = JCM 1125	A0A0R1YGI6	216
1423820	*Lactobacillus aviarius* subsp. *araffinosus* DSM 20653	A0A0R1ZSA7	365
1423727	*Lactobacillus brantae* DSM 23927	A0A0R2B010	363
		A0A0R2B0A6	346
1423729	*Lactobacillus cacaonum* DSM 21116	A0A0R2CW19	360
1423730	*Lactobacillus camelliae* DSM 22697 = JCM 13995	A0A0R2ERE5	362
		A0A0R2F552	360
1122146	*Lactobacillus ceti* DSM 22408	A0A0R2KJ20	363
1423734	*Lactobacillus composti* DSM 18527 = JCM 14202	X0PU41	363
1423735	*Lactobacillus concavus* DSM 17758	A0A0R1W3V6	391
1185325	*Lactobacillus coryniformis* subsp. *coryniformis* CECT 5711	J3JB76	356
		J3JCC0	363
748671	*Lactobacillus crispatus* ST1	D5GY45	364
		D5GZ81	345
		D5H022	352
1423738	*Lactobacillus dextrinicus* DSM 20335	A0A0R2BHL7	365
1400520	*Lactobacillus fabifermentans* T30PCM01	W6T5Z8	363
		W6TBY3	363
1612	*Lactobacillus farciminis*	A0A0H4LFI1	345
		A0A0H4LHP9	361
		A0A0H4LQL8	362
1423747	*Lactobacillus fuchuensis* DSM 14340 = JCM 11249	A0A0R1RTV4	352
		A0A0R1RWR1	364
1423750	*Lactobacillus ghanensis* DSM 18630	A0A0R1VEP2	346
1007676	*Lactobacillus ginsenosidimutans*	A0A0H4QF39	348
1423754	*Lactobacillus hamsteri* DSM 5661 = JCM 6256	A0A0R1YEU2	353
		A0A0R1YFJ6	347
1122147	*Lactobacillus harbinensis* DSM 16991	A0A0R1X7N9	340
		A0A0R1XDB5	364
405566	*Lactobacillus helveticus* DPC 4571	A8YTK6	288
1423758	*Lactobacillus hominis* DSM 23910 = CRBIP 24.179	I7L771	363
525329	*Lactobacillus jensenii* JV-V16	D6S2F6	344
257314	*Lactobacillus johnsonii* NCC 533	Q74HC9	356
		Q74HL9	345

1423763	*Lactobacillus kalixensis* DSM 16043	A0A0R1UAV3	352
267818	*Lactobacillus kefiranofaciens*	A0A1G5UVH1	370
		A0A1G5X7P2	345
		A0A1G5WAB0	352
1423769	*Lactobacillus manihotivorans* DSM 13343 = JCM 12514	A0A0R1QH79	362
		A0A0R1RFU7	371
1218508	*Lactobacillus mellis*	A0A0F4L0G6	365
1423770	*Lactobacillus mindensis* DSM 14500	A0A0R1QM29	356
1291734	*Lactobacillus nasuensis* JCM 17158	A0A0R1JIN1	351
		A0A0R1JZ64	343
1423775	*Lactobacillus nodensis* DSM 19682 = JCM 14932 = NBRC 107160	A0A0R1KB17	348
		A0A0R1KB58	363
1423777	*Lactobacillus oeni* DSM 19972	A0A0R1MB04	367
		A0A0R1MF18	362
321967	*Lactobacillus paracasei* ATCC 334	Q033U5	366
		Q036P6	366
1423790	*Lactobacillus pasteurii* DSM 23907 = CRBIP 24.76	I7IYW9	342
		I7JY07	336
		I7KKV2	363
		I7LAD2	261
1423792	*Lactobacillus perolens* DSM 12744	A0A0R1N3K0	362
1590	*Lactobacillus plantarum*	A0A0M4RAV6	363
		A0A0M4SAG9	364
		A0A0M5M5C6	362
220668	*Lactobacillus plantarum* WCFS1	F9URN7	363
		F9UT47	364
		F9UUH6	362
		F9UUI8	351
449659	*Lactobacillus pobuzihii*	A0A0R2L1C3	351
		A0A0R2L8D6	363
1423796	*Lactobacillus rennini* DSM 20253	A0A0R2D3K4	363
		A0A0R2D8G5	342
314315	*Lactobacillus sakei* subsp. *sakei* 23K	Q38WT3	364
1293598	*Lactobacillus saniviri* JCM 17471 = DSM 24301	A0A0R2MPE1	356
		A0A0R2MXW5	362
1423801	*Lactobacillus satsumensis* DSM 16230 = JCM 12392	A0A0R1UZE1	361
81857	*Lactobacillus selangorensis*	A0A0R2G744	363
1291052	*Lactobacillus sharpeae* JCM 1186 = DSM 20505	A0A0R1ZIU1	351
		A0A0R1ZX01	371
1231336	*Lactobacillus shenzhenensis* LY-73	U4TPC8	363
		U4TZH2	341
97137	*Lactobacillus* sp. ASF360	N1ZM26	341
1545702	*Lactobacillus* sp. wkB8	A0A089Y2K6	365
		A0A089Y639	351
		A0A089ZL45	364

1423810	*Lactobacillus thailandensis* DSM 22698 = JCM 13996	A0A0R2C675	334
1133569	*Lactobacillus vini* DSM 20605	A0A0R2C441	360
942150	*Lactobacillus xiangfangensis*	A0A0R2M1D1	363
		A0A0R2MKD9	346
420890	*Lactococcus garvieae* Lg2	F9VCI5	353
		F9VDF4	365
272623	*Lactococcus lactis* subsp. *lactis* Il1403	Q9CGG7	353
		Q9CJ33	364
297352	*Lactococcus piscium* MKFS47	A0A0D6DXT8	364
		A0A0D6E077	367
1215915	*Lactococcus raffinolactis* 4877	I7LNF6	363
		I7LPM3	299
		I7LQD5	353
596323	*Leptotrichia goodfellowii* F0264	D0GMP0	362
		D0GNG4	358
634994	*Leptotrichia hofstadii* F0254	C9MWS7	292
1785996	*Leptotrichia* sp. oral taxon 847	A0A0X8JUT5	363
1265818	*Listeria aquatica* FSL S10-1188	W7AWB9	362
1265820	*Listeria cornellensis* FSL F6-0969	W7BQE4	361
		W7BSB9	362
1081736	*Listeria fleischmannii* subsp. *coloradonensis*	H7F450	375
		H7F7U1	352
1265817	*Listeria floridensis* FSL S10-1187	W7C960	362
525367	*Listeria grayi* DSM 20601	D7UU60	360
		D7UW40	368
		D7V074	353
169963	*Listeria monocytogenes* EGD-e	Q8Y6G8	362
1265846	*Listeria rocourtiae* FSL F6-920	W7D3V2	362
		W7D7L0	235
		W7DQM0	361
1497679	*Listeriaceae bacterium* FSL A5-0209	A0A099W1R7	353
		A0A099W4B5	362
		A0A099WK82	361
1293441	*Lysinibacillus contaminans*	A0A0M8Q8B7	360
1220589	*Lysinibacillus odysseyi* 34hs-1 = NBRC 100172	A0A0A3IF92	360
1145276	*Lysinibacillus varians*	X2GVB2	360
1122219	*Megasphaera cerevisiae* DSM 20462	A0A0J6WZP9	360
1064535	*Megasphaera elsdenii* DSM 20460	G0VN95	360
706434	*Megasphaera micronuciformis* F0359	E2ZBK6	359
1111454	*Megasphaera* sp. BV3C16-1	U7UVK7	368
537288	*Megasphaera* sp. DJF_B143	A0A124EFZ1	360
1603888	*Megasphaera* sp. MJR8396C	A0A133SGN2	360
940190	*Melissococcus plutonius* ATCC 35311	F3Y8J1	353
		F3YB62	363
1344956	*Microbacterium* sp. TS-1	U2XPM0	360
500635	*Mitsuokella multacida* DSM 20544	C9KL95	377

36809	*Mycobacteroides abscessus*	A0A0U1AD61	363
		A0A0U1AK52	362
		A0A0U1AZR6	369
1034808	*Mycoplasma anatis* 1340	F9QDW7	363
29556	*Mycoplasma gallinaceum*	A0A0D5ZJ39	362
1048830	*Mycoplasma iowae* 695	G4EL66	372
221109	*Oceanobacillus iheyensis* HTE831	Q8ESL1	357
545501	*Oceanobacillus oncorhynchi*	A0A0A1M9C7	362
		A0A0A1MUU9	357
203123	*Oenococcus oeni* PSU-1	Q04GV5	361
1125712	*Olsenella profusa* F0195	U2TIP4	366
		U2UUL8	361
		U2V5M7	365
697284	*Paenibacillus larvae* subsp. *larvae* DSM 25430	V9WA07	302
1073571	*Paenibacillus riograndensis* SBR5	A0A0E4CYF2	349
1227352	*Paenibacillus* sp. FSL R7-277	W4CNH8	350
1505	*Paeniclostridium sordellii*	A0A0A1SIA6	363
1292036	*Paeniclostridium sordellii* ATCC 9714	T0CNG7	206
1220594	*Parageobacillus caldoxylosilyticus* NBRC 107762	A0A023DJV8	355
153151	*Parageobacillus toebii*	A0A150N081	356
862514	*Pediococcus acidilactici* DSM 20284	E0NG57	365
		E0NHJ0	371
480391	*Pediococcus argentinicus*	A0A0R2NA43	363
319652	*Pediococcus cellicola*	A0A0R2IQE3	365
		A0A0R2IUE5	361
701521	*Pediococcus claussenii* ATCC BAA-344	G8PBE2	362
51663	*Pediococcus damnosus*	A0A0R2HFB0	361
		A0A2G9VFL4	356
278197	*Pediococcus pentosaceus* ATCC 25745	Q03HJ1	363
484770	*Pelosinus* sp. UFO1	A0A075KAY3	363
1655638	*Peptococcaceae bacterium* 1109	A0A0J1DKH8	374
999898	*Peptococcaceae bacterium* CEB3	A0A0J1FGG8	366
658445	*Photobacterium gaetbulicola* Gung47	A0A0C5WQ52	365
1526927	*Planococcus* sp. PAMC 21323	A0A0B4R8G0	350
1385512	*Pontibacillus litoralis* JSM 072002	A0A0A5G0J7	361
411467	*Pseudoflavonifractor capillosus* ATCC 29799	A6NTC6	351
		A6NYD7	368
1671366	*Ruminococcus* sp. DSM 100440	A0A150Y881	362
526218	*Sebaldella termitidis* ATCC 33386	D1AG48	342
		D1AK78	361
		D1APW7	363
927704	*Selenomonas ruminantium* subsp. *lactilytica* TAM6421	I0GRB2	356
		I0GTZ3	357
546271	*Selenomonas sputigena* ATCC 35185	F4EUM5	361
706433	*Solobacterium moorei* F0204	E7MMB3	349
		E7MQV6	362

1276258	*Spiroplasma apis* B31	V5RHY0	368
1276246	*Spiroplasma culicicola* AES-1	W6A6J9	366
		W6A6T4	366
		W6A7P9	368
1276221	*Spiroplasma diminutum* CUAS-1	S5LZ98	367
		S5M045	372
216942	*Spiroplasma litorale*	A0A0K1W1G6	367
570509	*Spiroplasma melliferum* KC3	A0A037UPA3	369
838561	*Spiroplasma mirum* ATCC 29335	W0GP22	209
2138	*Spiroplasma poulsonii*	A0A2P6FEQ4	263
1276257	*Spiroplasma sabaudiense* Ar-1343	W6AA03	363
		W6AAV3	367
		W6AKH8	366
		W6AKP1	368
1276229	*Spiroplasma syrphidicola* EA-1	R4U566	372
		R4U6S7	346
1069536	*Sporolactobacillus inulinus* CASD	A0A0U1QNE3	359
1476	*Sporosarcina psychrophila*	A0A127VUS9	359
		A0A127W2H8	355
1413510	*Staphylococcus aureus* C0673	A0A033UGY8	365
		A0A033UPE9	349
93061	*Staphylococcus aureus* subsp. *aureus* NCTC 8325	Q2G1G7	346
367830	*Staphylococcus aureus* subsp. *aureus* USA300	A0A0H2XHV5	351
396513	*Staphylococcus carnosus* subsp. *carnosus* TM300	B9DJX5	354
176279	*Staphylococcus epidermidis* RP62A	Q5HLT4	350
28035	*Staphylococcus lugdunensis*	A0A133QAT3	349
1229783	*Staphylococcus massiliensis* S46	K9ANW1	348
904314	*Staphylococcus pettenkoferi* VCU012	H0DFP6	349
342451	*Staphylococcus saprophyticus* subsp. *saprophyticus* ATCC 15305	Q49ZN5	349
1262969	*Staphylococcus* sp. CAG:324	R6T6U8	345
553483	*Steptococcus equi* subsp. *zooepidemicus* H70	C0MDB8	365
519441	*Streptobacillus moniliformis* DSM 12112	D1AYQ8	338
1154860	*Streptococcus agalactiae* LMG 14747	V6YYS5	364
1273539	*Streptococcus iniae* IUSA1	V4QPY2	361
862967	*Streptococcus intermedius* B196	T1ZC60	351
1811193	*Streptococcus pantholopis*	A0A172Q7B0	364
301447	*Streptococcus pyogenes* serotype M1	Q99Z98	364
1777879	*Streptococcus* sp. DD11	A0A139P952	363
904294	*Streptococcus* sp. oral taxon 056 str. F0418	F9HIJ1	363
391295	*Streptococcus suis* 05ZYH33	A4VXQ7	283
		A4VY54	369
218495	*Streptococcus uberis* 0140J	B9DU07	362
273057	*Sulfolobus solfataricus* P2	Q97YQ2	335
273063	*Sulfurisphaera tokodaii* str. 7	Q973N4	344
1209989	*Tepidanaerobacter acetatoxydans* Re1	F4LRX5	361
224999	*Tepidanaerobacter syntrophicus*	A0A0U9HJR7	362

586416	*Terribacillus aidingensis*	A0A075LNS0	350
945021	*Tetragenococcus halophilus* NBRC 12172	G4L492	358
		G4L7Y6	363
1302648	*Tetragenococcus muriaticus* 3MR10-3	A0A091BZM9	360
		A0A091BZZ2	215
644966	*Thermaerobacter marianensis* DSM 12885	E6SHP2	377
1535750	*Thermoactinomyces* sp. Gus2-1	A0A094JKW4	361
2026	*Thermoactinomyces vulgaris*	A0A0N0YA19	364
1094508	*Thermoanaerobacterium saccharolyticum* JW/SL-YS485	I3VRJ7	360
		I3VRK3	363
1321815	*Treponema lecithinolyticum* ATCC 700332	U2M3T0	348
754027	*Treponema phagedenis* F0421	E7NSM7	345
36087	*Trichuris trichiura*	A0A077ZK10	626
1408226	*Vagococcus lutrae* LBD1	V6Q5P2	363
		V6Q6J4	371
1462526	*Virgibacillus massiliensis*	A0A024QGD1	347
1473	*Virgibacillus pantothenticus*	A0A0L0QKK4	365
		A0A0L0QN84	360
		A0A0L0QPQ2	356
1227360	*Viridibacillus arenosi* FSL R5-213	W4ERI8	365
137591	*Weissella cibaria*	A0A0D1LJQ4	365
1127131	*Weissella confusa* LBAE C39-2	H1X9R4	365
1777865	*Weissella* sp. DD23	A0A139QLE2	365
994573	*Youngiibacter fragilis* 232.1	V7I739	348

References

Bae, T., and Schneewind, O. (2006). Allelic replacement in *Staphylococcus aureus* with inducible counter-selection. *Plasmid* 55**,** 58-63.

Borisova, M., Gaupp, R., Duckworth, A., Schneider, A., Dalügge, D., Mühleck, M., Deubel, D., Unsleber, S., Yu, W., Muth, G., Bischoff, M., Götz, F., and Mayer, C. (2016). Peptidoglycan recycling in Gram-positive bacteria is crucial for survival in stationary phase. *mBio* 7**,** e00923-00916.

Ebner, P., Prax, M., Nega, M., Koch, I., Dube, L., Yu, W., Rinker, J., Popella, P., Flötenmeyer, M., and Götz, F. (2015). Excretion of cytoplasmic proteins (ECP) in *Staphylococcus aureus*. *Mol Microbiol* 97**,** 775-789.

Fey, P.D., Endres, J.L., Yajjala, V.K., Widhelm, T.J., Boissy, R.J., Bose, J.L., and Bayles, K.W. (2013). A genetic resource for rapid and comprehensive phenotype screening of nonessential *Staphylococcus aureus* genes. *MBio* 4**,** e00537-00512.

Geiger, T., Francois, P., Liebeke, M., Fraunholz, M., Goerke, C., Krismer, B., Schrenzel, J., Lalk, M., and Wolz, C. (2012). The stringent response of *Staphylococcus aureus* and its impact on survival after phagocytosis through the induction of intracellular PSMs expression. *PLoS Pathog* 8**,** e1003016.

Gibson, D.G., Young, L., Chuang, R.Y., Venter, J.C., Hutchison, C.A., 3rd, and Smith, H.O. (2009). Enzymatic assembly of DNA molecules up to several hundred kilobases. *Nat Methods* 6**,** 343-345.

Hanahan, D. (1983). Studies on transformation of *Escherichia coli* with plasmids. *J Mol Biol* 166**,** 557-580.

Löfblom, J., Kronqvist, N., Uhlén, M., Ståhl, S., and Wernérus, H. (2007). Optimization of electroporation-mediated transformation: *Staphylococcus carnosus* as model organism. *J Appl Microbiol* 102**,** 736-747.

Monk, I.R., Shah, I.M., Xu, M., Tan, M.W., and Foster, T.J. (2012). Transforming the untransformable: application of direct transformation to manipulate genetically *Staphylococcus aureus* and *Staphylococcus epidermidis*. *MBio* 3.

Studier, F.W., and Moffat, B.A. (1986). Use of bacteriophage T7 RNA polymerase to direct selective high-level expression of cloned genes. *Mol. Biol.* 189**,** 113-130.
